# G-Quadruplex–specific action of chloroquine-based immunomodulator drugs to inhibit the cancer progression

**DOI:** 10.1016/j.jbc.2025.110753

**Published:** 2025-09-22

**Authors:** Sunipa Sarkar, Akash Chatterjee, Subhojit Paul, Asim Bisoi, Prosenjit Sen, Prashant Chandra Singh

**Affiliations:** 1 School of Chemical Sciences, Indian Association for the Cultivation of Science, Jadavpur, Kolkata, India; 2 School of Biological Sciences, Indian Association for the Cultivation of Science, Jadavpur, Kolkata, India

**Keywords:** downregulation, drugs, G-quadruplex, immunomodulator, cancer, oncogene

## Abstract

Immunomodulatory drugs, particularly hydroxychloroquine (HCQ) and chloroquine are in the preclinical investigation for cancer therapy, along with their extensive application in autoimmune and parasitic diseases. A hallmark of cancer cells is the elevated expression of oncogenes that drive tumor progression, often regulated by G-quadruplex (G4) DNA structures located within their upstream promoter regions. This study elucidates that HCQ stabilizes the cellular G4 landscape most efficiently compared to other quinoline-based immunomodulatory drugs within oncogenic DNA, particularly the c-myc oncogene, a pivotal regulator of cancer progression. The drug-induced stabilization of c-myc G4 correlates with significant suppression of its transcriptional activity, culminating in a reduction of invasion and migration of triple-negative breast cancer cells. Mechanistically, the strong electrostatic interaction between the G4 phosphate backbone and the drug's charged side chain, anchors its quinoline group to enhance stacking with loop and quartet regions, stabilizing the G4. The *in vivo* investigation unveils the HCQ's capacity to potentiate the efficacy of conventional chemotherapeutic agents, representing it as a plausible candidate for adjunctive therapy. This study depicts an unconventional anticancer mechanism of immunomodulator drugs, wherein it exerts preferential transcriptional repression of the c-myc oncogene through G4-dependent stabilization, unveiling a novel strategy in oncological intervention.

The prevalence of cancer constitutes a formidable threat to human health, with chemotherapy comprising a frontline therapeutic intervention (1). However, extant chemotherapeutic regimens frequently engender significant side effects and can elicit acquired resistance, precipitating disease relapse (1). The elucidation of innovative chemotherapeutic targets and the development of approaches to overcome treatment resistance are of utter importance to enhance therapeutic efficacy and clinical outcomes among cancer patients (1, 2). Oncogenesis is intricately linked to genetic aberrations in regulatory genes, notably the aberrant expression of various oncogenes that propagate dysregulated signals, leading to cell proliferation, migration, increased invasion into the surrounding matrix, etc (2, 3). c-myc emerges as a pivotal oncogene, exhibiting upregulation in approximately 70% of cancer instances (4, 5). Directly addressing the overexpressed c-myc protein poses a formidable challenge owing to the absence of viable small molecule binding sites and its limited lifespan (6). Consequently, efforts have been redirected toward the exploration of alternative avenues in cancer treatment, focusing on targeting specific DNA secondary structures associated with oncogenes, with particular emphasis on the extensively overexpressed c-myc (7, 8, 9, 10).

G-quadruplexes (G4s) are secondary DNA structures formed by the folding of guanine-rich DNA sequences in the presence of monovalent cations (11). G4 structures are stabilized through the Hoogsteen hydrogen bond and π stacking between the guanine nucleobases. Several genomic sequences having guanine-rich domains are found to be more thermodynamically stable than the dsDNA and these sequences can directly form G4 structure (8, 12). The formation of G4 structures has been identified in the promoter region of several oncogenes through experimental and computational studies (8, 9, 13, 14, 15). Stabilizing these G4 structures within oncogene promoters has been demonstrated to reduce transcriptional rates, offering a novel avenue for cancer therapy (16, 17, 18, 19). Despite concerted efforts to develop small molecule ligands (20) capable of stabilizing G4 structures within oncogene promoter regions, no compounds have yet attained clinical approval as targeted antineoplastic agents, likely because of the concerns about their compatibility with biological systems and poor pharmacological properties (21, 22, 23, 24, 25, 26). Therefore, it is imperative to investigate the gene regulatory effects of the drug candidates currently undergoing clinical trials, specifically their interactions with G4 structures. Such investigations are crucial as these molecules hold potential as viable candidates for future cancer therapies.

Hydroxychloroquine (HCQ, Fig. 1*A*) and chloroquine (CQ, Fig. 1*A*) are important immunomodulatory drugs that have been extensively used for the treatment of rheumatoid arthritis, systematic lupus erythematosus, and malaria (27, 28, 29). Recently, it has been reported that HCQ and CQ may affect the cancer cells and may increase the tumor sensitivity to existing cancer treatments (30, 31). Moreover, numerous clinical trials have been initiated to evaluate the therapeutic potential of both HCQ and CQ as novel targeted agents against a diverse array of cancer histologies, with over 30 studies currently registered across all phases of clinical evaluation (31, 32). There are few studies in which the plausible action of HCQ and CQ on different cancer cell lines have been discussed (33, 34, 35, 36, 37). Inhibition of autophagy is one of the most studied anticancer actions of HCQ and CQ (33). It has been proposed that these drugs get protonated upon entering lysozyme which gets them trapped in the acidic lysosome and inhibits the lysosomal degradative enzymes (33). However, recent studies suggest that CQ seems to transiently perturb lysosome integrity and function rather than suppressing an integrated autophagy process as proposed earlier (34). The other few proposed actions of the CQ are the inhibition of kappa B and CXCL2/CXCl4 signaling pathways (35, 36, 37). Apart from these studies, the role of HCQ in tumor immunotherapy has been investigated; however, it has been proposed that HCQ decreases the benefit of anti-PD1 immune checkpoint blockade in tumor immunotherapy (38, 39). Hence, it can be speculated that the antineoplastic efficacy of immunomodulatory drugs HCQ and CQ may have another selective biological target. While a limited number of studies have explored the putative antineoplastic efficacy of HCQ and CQ across various cancer cell lines, the precise molecular mechanisms underpinning their activity remain incompletely defined (33, 34, 35, 36, 37, 38, 39).

**Figure 1:**
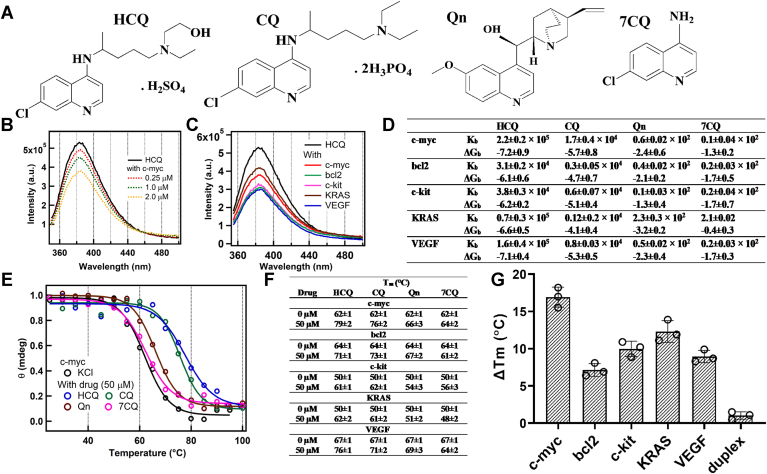
**Binding and Stability of G4-DNA in presence of drugs.***A*, the chemical structure of drug molecules HCQ, CQ, and their structural analogs Qn and 7CQ. *B*, the emission spectra of HCQ (10 μM) in buffer and different concentrations of c-myc DNA. *C*, the emission spectra of HCQ (10 μM) in buffer and different G4 DNA sequences. *D*, the values of binding constant (K_b_, M^−1^) and binding free energy (ΔG_b,_ kcal/mole) of drugs with gene sequences at 25 °C. The correlation coefficient of the linear regression (R^2^) of the fitted data was in the range of 0.95 to 0.99. *E*, the melting curve of the 5 μM c-myc in 2 mM KCl and the presence of different drug molecules (50 μM). *F*, the melting temperature (T_m_) of the G4 formed from the different G4 sequences and the effect of the drug molecules on the melting temperature of these sequences. *G*, the change in melting temperature (ΔT_m_ = T_m_ of G4 in drug-T_m_ of G4 in KCl) of different G4 sequences in the presence of HCQ and its comparison with the duplex DNA sequences. The error bars indicate the mean ± SD of three independent melting experiments. CQ, chloroquine; G4, G-quadruplex; HCQ, hydroxychloroquine.

Recently, the possibility of the binding of HCQ with G4 has been illustrated by our group using the spectroscopic and molecular simulation methods (40). However, despite the evident therapeutic imperative to selectively target aberrantly overexpressed oncogenes, the antineoplastic efficacy of immunomodulatory agents against such targets remains largely unexplored. Elucidating the precise molecular mechanisms by which this class of pharmacologic impacts overactivated oncogenic signaling cascades would augment their clinical development as targeted chemotherapeutics. In this study, we have established that immunomodulators, HCQ, and CQ significantly reduced the transcriptional activity of the c-myc predominantly stabilizing the G4 in upstream of the promoter region of the gene and significantly restricting the progression of cancer. This approach represents a promising alternative strategy to improve clinical outcomes in this aggressive breast cancer subtype.

## Results

### Binding and stability of G4-DNA sequences of different genes with HCQ and its analogs

Figure 1*B* illustrates the emission spectra of HCQ in buffer, with varying concentrations of G4 DNA sequence of the promoter region of c-myc obtained from the excitation at the isosbestic point of the HCQ and DNA (295 nm, Fig. S1). The observed successive decrease in the fluorescence intensity of HCQ following the addition of c-myc G4 DNA suggests a plausible interaction between HCQ and the G4 DNA sequence of the c-myc gene, in line with the earlier finding that the binding of HCQ with DNA results in the quenching of its fluorescence (40). The fluorescence of HCQ exhibited a decrement upon the addition of other G4-forming DNA sequences (bcl2, c-kit, KRAS, and VEGF) concurrently with c-myc (Fig. 1*C*). This observation suggests that HCQ may interact with all these G4-forming DNA sequences. The fluorescence data for other quinoline derivative molecules CQ, Qn, and 7CQ similarly demonstrate a reduction in fluorescence intensity upon the addition of these G4-forming sequences (Fig. S2). Notably, HCQ and CQ manifest a more pronounced reduction in fluorescence intensity upon the addition of G4 DNA sequences compared to their respective analogs, Qn and 7CQ. This observation implies that the binding propensities of the drugs HCQ and CQ with the G4 DNA sequences may differ from their analogs Qn and 7CQ. Therefore, the determination of binding constants (K_b_) and binding free energy (ΔG_b_) for all the molecules interacting with different G4-forming DNA sequences was executed through the assessment of fluorescence changes induced by the addition of DNA sequences and utilizing the modified Stern-Volmer method (Equation 1), as depicted in Figures 1*D* and S3. The calculated K_b_ and ΔG_b_ values indicate that the binding affinity of HCQ and CQ for any G4-forming DNA sequence is relatively higher than Qn and 7CQ. The binding affinity of HCQ and CQ to G4-forming DNA sequences (in the order of ∼10^5^–10^4^) is lower than that of several well-known ligands, which exhibit binding constants in the range of ∼10^6^ (41). The structures of HCQ and CQ closely resemble that of 7CQ, differing primarily in the presence of charge on the nitrogen of the ring and the tertiary nitrogen at the terminal of the side chain. In contrast, Qn shares the quinoline ring structure but features distinct substitutions at the fourth and seventh positions compared to other synthetic analogs. Moreover, Qn predominantly exists in its deprotonated form at pH 7.4. Structural and binding correlation analysis suggests that the charge and side chain of HCQ and CQ are crucial for facilitating their interaction with G4 DNA sequences.

To assess the impact of drug molecules on the structural aspects of G4, CD measurements were performed for all G4 DNA sequences in both the absence and presence of drugs (Fig. S4). Consistent with prior studies, CD spectra of G4 DNA structures formed in 2 mM KCl displayed positive ellipticity peaks at 265 nm and negative ellipticity at 245 nm, indicative of the parallel conformation of the G4 (42). The addition of drug molecules resulted in marginal changes in the CD peak ellipticity without the emergence of new peaks, suggesting that drug binding does not induce significant structural alteration in the parallel G4 conformation across all sequences. For a quantitative assessment of drug-induced thermal stability of G4, the melting temperature (T_m_) of G4 was determined in the absence and presence of drugs by monitoring temperature-dependent changes in the characteristic 265 nm CD peak of the G4 (Figs. 1, *E* and *F*, and S5). The data revealed a notable increase in the T_m_ of G4-forming DNA sequences, particularly with HCQ and CQ (11–17 °C), in contrast to Qn and 7CQ (0–5 °C). This trend aligns with the binding data and emphasizes the crucial role of both the charge and side chain of HCQ and CQ in G4 stabilization. On the other hand, the presence of HCQ and CQ has minimal impact on the T_m_ of duplex DNA, indicating the selective nature of G4 stabilization by these drug molecules (Fig. 1*G*). Noticeably, the HCQ/CQ-induced stability of G4 structures exhibited sequence dependency, with the highest change in melting temperature (ΔT_m_) for c-myc (∼17 °C) compared to other G4-forming sequences such as bcl2 (7 °C), c-kit (11 °C), KRAS (12 °C), and VEGF (9 °C). The T_m_ data reveal that HCQ and CQ stabilize selectively to G4 in the promoter regions of various genes, with the greatest stabilization observed for the c-myc G4 DNA compared to other examined promoter sequences.

Further, the competitive FRET-melting assay of the c-myc attached with donor fluorescein amidites (6-FAM at 5′end) and acceptor carboxy tetramethylrhodamine (TAMRA at the 3′end) was performed in KCl, HCQ, the different competitive G4 and duplex DNA sequences in 1:1 and 1:10 ratio than c-myc. The T_m_ of c-myc in KCl obtained from the FRET data increases by ∼12 °C with the addition of HCQ which is in agreement with the CD data (Fig. S6*A*). The competitive FRET-melting data of c-myc with HCQ does not change appreciably with the addition of other G4 and duplex DNA sequences in a 1:1 ratio indicating that HCQ prefers to bind with the c-myc than other G4 and duplex DNA sequences (Fig. S6*B*). Indeed, the T_m_ of c-myc with HCQ does not change appreciably even in the presence of a higher ratio of G4 DNA sequences (Fig. S6*C*) than the c-myc (10:1). The competitive FRET-melting assay and CD melting data indicate that the stability of the G4 by the HCQ and CQ is reasonably selective to the c-myc than other G4 sequences.

### Cellular visualization of G4 landscape in the presence of HCQ

**Figure 2:**
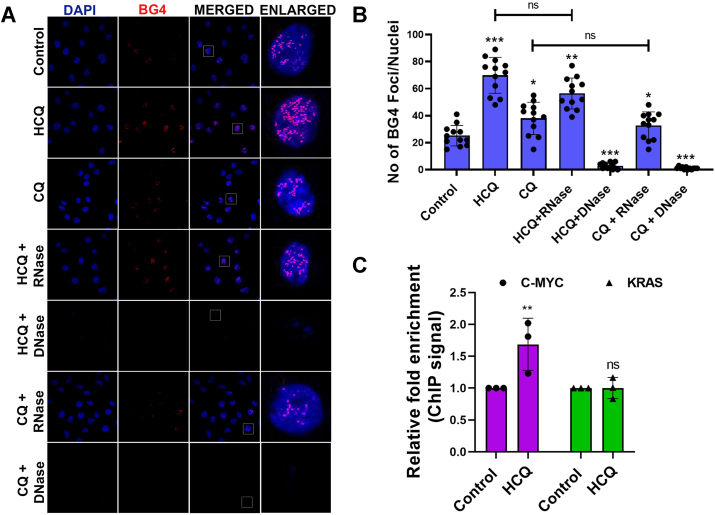
**Cellular Visualization of G4 Landscape in presence of HCQ and CQ.***A*, immunofluorescence images stained for nucleus (DAPI, *blue*), G4 structures showing BG4 foci (*red*), and merged images in the absence (control) and the presence of HCQ and CQ alone as well as along with RNase and DNase, respectively using MDA-MB-231 breast cancer cell line. *B*, quantification of BG4 stained foci was performed using ImageJ and represented as a *bar graph*. *C*, ChIP-qPCR data showing relative enrichment of G4 structure at the c-myc and KRAS gene in the vehicle and HCQ-treated cells. The error bars indicate the mean ± SD of three independent experiments and the statistical significance levels denoted as follows: ns = not significant, *p* > 0.05, ∗*p* < 0.05, ∗∗*p* < 0.01, ∗∗∗*p* < 0.001, and ∗∗∗∗*p* < 0.0001, assessed using ANOVA with Tukey *post hoc* test for multiple comparisons. ChIP-qPCR, chromatin immunoprecipitation–quantitative PCR; CQ, chloroquine; DAPI, 4′,6-diamidino-2-phenylindole; HCQ, hydroxychloroquine.

Our *in vitro* experiments suggest that HCQ stabilizes the G4 structure preferably within the c-myc promoter region compared to other genes. Hence, first, we assessed the toxicity effect of drugs by performing cell viability assays using human embryonic kidney (HEK) 293 cells, a noncancerous HEK-derived cell line, and MDA-MB-231 cells, a model of triple-negative breast cancer (TNBC). Treatment with HCQ at concentrations of 50 to 100 μM resulted in a marked reduction in the viability of MDA-MB-231 cells, while inducing minimal cytotoxicity in HEK293 cells. In comparison, CQ and 7CQ were less effective in reducing MDA-MB-231 cell viability and did not exhibit significant cytotoxic effects on HEK293 cells (Fig. S7). These findings suggest that HCQ exhibits greater selective cytotoxicity toward cancer cells than normal cells. The cell viability data indicates that ∼50 to 100 μM of HCQ shows the most significant decrease in the cell viability, hence, further all the cellular experiments have been performed in the same concentration range of HCQ (Fig. S7). To substantiate whether HCQ specifically stabilizes G4 in the cellular system, we employed the G4-specific antibody BG4 (43), a well-characterized probe for the determination of G4 landscape in the MDA-MB-231 breast cancer cell line. Upon HCQ treatment, the immunofluorescence image revealed numerous, intense BG4-stained nuclear foci compared to vehicle control, suggesting an enhanced stabilization of G4 structures within the nucleus by HCQ (Fig. 2, *A* and *B*). To distinguish whether these BG4 foci corresponded specifically to G4 structures within DNA rather than RNA, we conducted follow-up experiments using DNase and RNase treatments before BG4 staining. The RNase treatment left the nuclear distribution of BG4 foci largely unchanged, indicating that the observed G4 structures were mostly not associated with RNA. However, DNase treatment markedly diminished the nuclear BG4 signal, leading to a significant reduction in BG4 foci (Fig. 2, *A* and *B*). This observation supports the conclusion that HCQ mainly stabilizes G4 within DNA, while G4 structures within RNA remain almost unaffected by HCQ. The immunofluorescence data of nuclear G4 foci in cells treated with CQ indicate that CQ also stabilized the DNA G4. However, the CQ was found to be comparatively less effective than HCQ in stabilizing G4, as the number of G4 foci per nucleus was higher in HCQ-treated cells than CQ-treated cells. In contrast to HCQ and CQ, 7CQ treatment did not have any noticeable impact on G4 foci in the cancer cells pointing out the incapability of the binding of 7CQ with G4 of DNA (Fig. S8). All these observations combinedly indicate the possible involvement of the charged side chain and the OH group of HCQ in the stabilization of the G4 in the cellular system.

To further validate the preferable stabilization of the G4 within different genes, we performed chromatin immunoprecipitation followed by quantitative PCR (ChIP-qPCR) using the G4-specific BG4 antibody for c-myc and KRAS (Fig. 2*C*). Both c-myc and KRAS were chosen for the experiments as HCQ enhances the T_m_ of the G4 of these two sequences maximally compared to other sequences used in this study. The ChIP-qPCR analysis revealed a pronounced enrichment of BG4 binding at the c-myc gene following HCQ treatment, indicating a marked stabilization of the G4 structure. In contrast, the BG4 signal at the KRAS gene, a region also capable of forming G4 structures but not specifically targeted by HCQ, remained low and unaffected by HCQ treatment, revealing the preference of HCQ for the c-myc G4 in the cellular system. The KRAS-G4 contains a greater number of nucleobases in its loop regions than the c-myc. This structural difference may influence the binding affinity of HCQ to these sequences which can cause the differential enrichment observed in BG4 ChIP-qPCR assays. Notably, a previous study demonstrated that variations in loop length between KRAS and c-myc G4s result in a reduced cavity size in the KRAS G4, thereby diminishing ligand binding relative to c-myc (44).

In a recent study, G4 DNA structures have been shown to act as positive regulators of c-myc transcription under basal physiological conditions (45). Hence, to investigate whether transcription factors (TFs) compete with HCQ for binding to c-myc G4 binding region, thereby potentially leading to transcriptional inhibition, we performed ChIP-qPCR analysis using an antibody against SP1, a known TF that binds to the c-myc promoter (45). Our data revealed that HCQ treatment significantly reduced SP1 enrichment at the c-myc promoter, while no significant change in SP1 binding was observed at the KRAS promoter (Fig. S9). This suggests that HCQ binding with c-myc G4 may hinder TF binding, thereby contributing to the observed downregulation of c-myc expression. Overall, our data indicate that HCQ prefers to bind and stabilize the G4 structure within the c-myc gene compared to other quinoline derivative immunomodulator drugs, as well as affect the TFs enrichment on the c-myc gene, leading to its transcriptional suppression.

### HCQ-mediated selective stabilization of G4 and transcriptional repression of oncogenes in cancer cells

**Figure 3:**
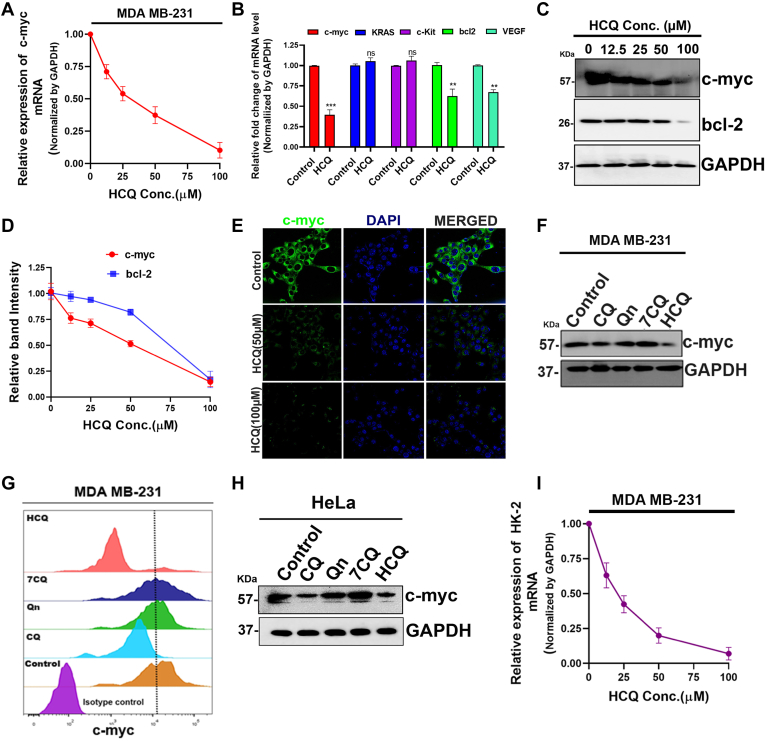
**HCQ suppresses oncogene expression.***A*, qRT-PCR analysis of c-myc mRNA level following different doses of HCQ treatment for 48 h in MDA-MB-231 cells. GAPDH was used as the normalization control. *B*, qRT-PCR analysis of c-myc, KRAS, c-kit, bcl-2, and VEGF mRNA levels following 50 μM HCQ treatment for 48 h in MDA-MB-231 cells. GAPDH was used as the normalization control. *C* and *D*, Western blot analysis of c-myc and bcl-2 in MDA-MB-231 cells following HCQ treatments with different doses for 48 h. The band intensity was calculated using ImageJ and plotted as relative band intensity using GraphPad Prism8. *E*, assessment of expression levels of c-myc *via* immunofluorescence in MDA-MB-231 cells following a 48-h exposure to either 100 μM or 50 μM HCQ. Nuclei are counterstained in blue with DAPI. *F*, Western blot analysis of c-myc expression in MDA-MB-231 cell line after the indicated treatments. *G*, flow cytometry analysis of c-myc expression in MDA-MB-231 cells after the indicated treatments for 48 h. *H*, Western blot analysis of c-myc expression in HeLa cells following indicated treatments for 48 h. *I*, qRT-PCR analysis of HK-2 mRNA levels following dose-dependent HCQ treatment for 48 h in MDA-MB-231 cells. GAPDH was used as the normalization control. The error bars indicate the mean ± SD of three independent experiments and the statistical significance levels denoted as follows: ns = not significant, *p* > 0.05, ∗*p* < 0.05, ∗∗*p* < 0.01, ∗∗∗*p* < 0.001, and ∗∗∗∗*p* < 0.0001, assessed using ANOVA with Tukey *post hoc* test for multiple comparisons. CQ, chloroquine; DAPI, 4′,6-diamidino-2-phenylindole; HCQ, hydroxychloroquine; qRT-PCT, quantitative real-time PCR.

The biophysical and cellular experiments revealed pronounced binding and stabilization of c-myc G4 by HCQ. To determine the impact of HCQ binding to the G4 on gene regulation, quantitative real-time PCR analysis of MDA-MB-231 breast cancer cells treated with varying HCQ concentrations showed a significant reduction in c-myc mRNA levels. Notably, this reduction in c-myc mRNA level was observed even at low HCQ doses, with the most pronounced inhibition occurring at a concentration of 100 μM. These findings suggest that HCQ treatment is associated with a dose-dependent suppression of c-myc gene expression in MDA-MB-231 breast cancer cells, indicating a potential regulatory role for HCQ in modulating c-myc mRNA levels. (Fig. 3*A*). Assessment of other genes harboring G4 structures (KRAS, c-kit, bcl2, VEGF) revealed HCQ-induced transcriptional repression, particularly notable for c-myc compared to modest effects on other genes (Fig. 3*B*). Western blot analysis confirmed concentration-dependent decreases in c-myc protein levels following HCQ treatment, with a lesser impact on bcl2, requiring higher concentrations for comparable repression (Fig. 3, *C* and *D*). Immunofluorescence analysis also corroborated HCQ-mediated reductions in c-myc protein expression in MDA-MB-231 cells (Fig. 3*E*). Structure-activity relationship studies involving HCQ, CQ, Qn, and 7CQ exhibited marked suppression of c-myc expression by HCQ and CQ, while Qn and 7CQ had negligible effects, aligning with biophysical experiments and emphasizing the importance of charged side chain of HCQ in preferential interaction and stabilization of c-myc G4 (Figs. 3, *F* and *G*, S10).

Further analysis in human cervical cancer cells (HeLa) supported HCQ-induced c-myc suppression, suggesting a broad transcriptional inhibitory mechanism across cancer cell types (Figs. 3*H*, S10*C*). Although HCQ exhibited a slight decrease in bcl2 and VEGF levels, the extent was significantly less than that observed for c-myc. This decrease in bcl2 and VEGF transcriptional activity may be attributed to G4 stabilization or the indirect impact of reduced c-myc expression because c-myc is the designated transcription regulator for these two genes (46). *In vitro* G4 stabilization studies indicated that the effect of HCQ on bcl2 is less pronounced than on KRAS and c-kit, whereas, HCQ did not affect much transcriptional activity of KRAS and c-kit. Additional analysis of HK2, regulated by c-myc (47) without G4 structures, also showed a significant decrease in HK2 mRNA levels post-HCQ treatment (Fig. 3*I*). This raises the possibility that the reduction in bcl2 and VEGF transcriptional activity could be predominantly attributed to diminished c-myc expression directly influencing their transcription by binding to their promoters rather than G4 stabilization by HCQ. These findings emphasize HCQ's potent modulation of c-myc transcription with potential therapeutic implications.

### Anticancer activity of HCQ alone and in combination with doxorubicin in cell system and breast cancer murine model

**Figure 4:**
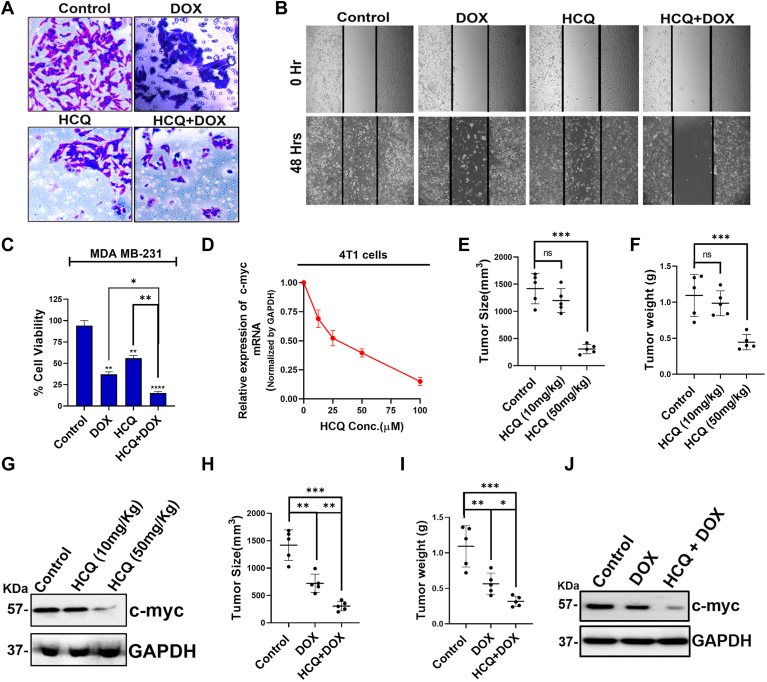
**Anticancer activity of HCQ.***A*, transwell invasion assay showing the extent of invasion of MDA-MB-231 cells after the indicated treatments. (HCQ 50 μM and DOX 1.5 μM). *B*, representative images of wound healing assay showing the extent of migration of MDA-MB-231 cells after the indicated treatments. (HCQ 50 μM and DOX 1.5 μM). *C*, impact of HCQ, DOX, and combination therapy for 72 h on the cell viability of MDA-MB-231 (human triple-negative breast cancer) cells. (HCQ 50 μM and DOX 1.5 μM). Each experiment was conducted in triplicates, and the data were analyzed and plotted using GraphPad Prism 8. *D*, qRT-PCR analysis of c-myc mRNA level following different doses of HCQ treatment for 48 h in 4T1 cells. GAPDH was used as the normalization control. *E* and *F*, statistical analysis of tumor size (*E*) and tumor weight (*F*) in Balb/c mice bearing tumor derived from 4T1 cells subjected to either control (saline) or HCQ (10 mg/kgbdwt or 50 mg/kgbdwt). *G*, Western blot analysis of c-myc expression in tumors from BALB/c mice following the sacrifice. GAPDH was used as a loading control. *H* and *I*, measurement of tumor size (*H*) and tumor weight (*I*) in Balb/c mice following exposure to control, DOX (5 mg/kgbdwt), and combination therapy of both HCQ and DOX from 4T1-derived tumors. *J*, Western blot analysis of c-myc expression in tumors following indicated treatments. The error bars indicate the mean ± SD of three independent experiments and the statistical significance levels denoted as follows: ns = not significant, *p* > 0.05, ∗*p* < 0.05, ∗∗*p* < 0.01, ∗∗∗*p* < 0.001, and ∗∗∗∗*p* < 0.0001, assessed using ANOVA with Tukey *post hoc* test for multiple comparisons. CQ, chloroquine; Dox, doxorubicin; HCQ, hydroxychloroquine; qRT-PCT, quantitative real-time PCR.

Previous research has underscored the significant role of c-myc in governing cancer cell migration and invasion (48). In line with these insights, our investigation aimed to decipher whether HCQ treatment in breast cancer MDA-MB-231 cells influences these critical cancer cell characteristics. Employing transwell invasion and wound healing assays, a substantial reduction in both invasion and migration of TNBC cells following HCQ treatment was observed. Concurrently, viability assessments demonstrated a decrease in breast cancer cell viability upon HCQ treatment compared to vehicle-treated cells (Fig. 4, *A*–*C*). Next, we explored the efficacy of HCQ, alone and in combination with doxorubicin (DOX), against breast cancer cells. Intriguingly, the combined treatment exhibited a more pronounced reduction in cell migration and invasion than either agent alone. MTT assay further unveiled a significant decrease in breast cancer cell viability with the DOX and HCQ combination, surpassing the effects observed with individual treatment. These findings hint at a potential additive interaction between HCQ and DOX in influencing breast cancer cell motility and survival, meriting further exploration in preclinical and clinical contexts. In summary, our study positions HCQ as a promising stand-alone agent for TNBC therapy through c-myc transcriptional downregulation and suggests its potential as an adjunctive in combination with chemotherapy (Fig. 4, *A*–*C*).

To unravel the *in vivo* antitumor mechanism of HCQ, we conducted a preliminary investigation utilizing mouse-specific TNBC cells 4T1. The study focused on assessing HCQ's impact on c-myc transcription levels, revealing a concentration-dependent downregulation akin to its effects on human TNBC cells (Fig. 4*D*). Subsequently, 4T1 cells were subcutaneously injected into Balb/c mice, and after tumor development, mice were treated with either a higher dose (50 mg/kgbdwt) or a lower dose (10 mg/kgbdwt) of HCQ. Results demonstrated a significant reduction in tumor volume and weight at the higher dose, while the lower dose proved ineffective. Western blot analysis indicated a substantial decrease in c-myc protein levels at 50 mg/kgbdwt, suggesting a potential dose threshold for antitumor efficacy (Fig. 4, *E*–*G*, S11). Interestingly, the lower HCQ dose, when combined with DOX, exhibited a significant reduction in tumor parameters compared to DOX alone, emphasizing potential adjunctive therapy benefits. Further, we analyzed the c-myc protein levels in tumor cells isolated from the treated mice (Fig. 4, *H*–*J*). A significant reduction in c-myc levels was observed following DOX treatment and the reduction in the c-myc level is even more pronounced when HCQ and DOX are administered together (Figs. 4, *H*–*J*, S11). To determine whether HCQ influences DOX uptake or efflux, we performed fluorescence microscopy to assess intracellular DOX accumulation in MDA-MB-231 cells treated with DOX in the presence or absence of HCQ. Our analysis revealed no detectable difference in intracellular DOX fluorescence intensity following HCQ treatment, indicating that HCQ does not alter cellular DOX uptake significantly (Fig. S12). These results suggest that the HCQ downregulates the c-myc transcription possibly by binding with the G4 sequence of the c-myc gene, which reduces tumor size and volume. This study indicates the potential of HCQ in the treatment of breast cancer either alone or adjunctive therapeutic manner.

### Binding process of c-myc G4 with HCQ using ^1^H-NMR spectroscopy and MD simulation

**Figure 5:**
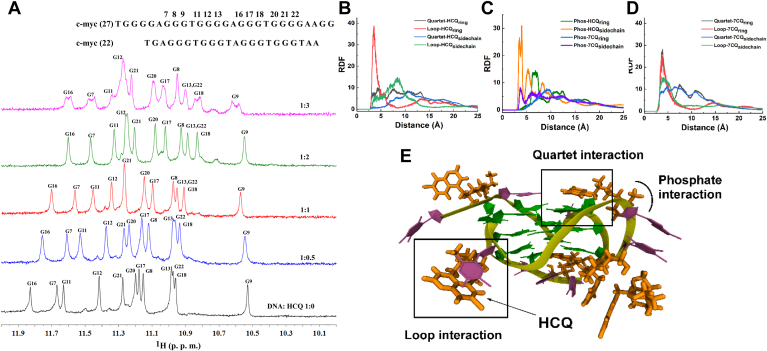
**Binding process of c-myc G4 with HCQ using NMR and MD simulation.***A*, ^1^H NMR spectra representing the imino region of the G4 DNA during the titration of c-myc with HCQ. The G4-imino protons of quartet and the ratio of c-myc and HCQ are labeled in each spectrum. The radial distribution function (RDF) around the quartet, loop, and phosphate backbone of the G4 with HCQ and 7CQ ring as well as a side chain are depicted in (*B*), (*C*), and (*D*), respectively. *E*, snapshots of the G4 structure surrounded with HCQ from the last frame of the 1 μs simulation. The G bases in the G4 structure are shown in *green color* and the A, and T bases in the loop regions are shown in *purple color*. HCQ has been shown in *orange color*. The stacking mode of interactions with G base in quartet and T base in loop regions were highlighted using a *square box* and the phosphate interaction is highlighted by a *line*. HCQ, hydroxychloroquine; CQ, chloroquine; G4, G-quadruplex.

The above-discussed data depicted that the binding of HCQ provides significant stability to the c-myc G4 and decreases the transcription of the c-myc. To further explore the plausible mechanism of HCQ binding to the c-myc G4, we conducted ^1^H-NMR experiments in the imino region (10–12 ppm) of the c-myc G4 (Fig. 5*A*). Consistent with earlier reports, the NMR spectra of c-myc G4 exhibited 12 well-resolved peaks, corresponding to the formation of three tetrads within the c-myc G4 (Fig. S13) (42, 44, 49). Upon the addition of HCQ, the fast chemical exchange of protons in the c-myc G4 was perturbed, resulting in the appearance of new sets of peaks, indicative of the formation of the HCQ–c-myc G4 complex. The most significant perturbations in the chemical shift of peaks were observed for G7, G11, and G16, which constitute the 5ʼ end of the c-myc G4, while interactions were less prominent with the 3ʼ end and the central tetrad of G4 (Fig. S13). Additionally, we conducted NMR experiments on the c-myc G4 with the addition of 7CQ (Fig. S13). The change in NMR peaks of the G tetrads in the presence of 7CQ was similar to that observed with HCQ. A comparative analysis of the change in chemical shift data for c-myc in the case of HCQ and 7CQ suggests that the quinoline group of HCQ is interacting predominately with the 5ʼ end of the c-myc G4 through stacking mode.

Next, the molecular dynamics (MD) simulation of the G4 structure of c-myc in water and the presence of HCQ and 7CQ has been performed for 1 μs to illustrate the binding of these drugs with G4. The time evolution of the RMSD values of the G4 structure in the absence and presence of drugs is almost constant with slight fluctuation (Fig. S14), indicating that the structure of G4 is intact on the binding of HCQ, in accordance with the CD data. The drug molecules, HCQ and 7CQ, possess two binding sites: one is defined as an aromatic ring having a quinoline ring (drug name_ring_) and the remaining part is considered as side chain (drug name_side chain_). To delve deeper into the binding modes, the radial distribution function (RDF) of these two binding sites of HCQ and 7CQ with quartet, loop, and phosphate backbone regions of G4 has been calculated (Fig. 5, *B*–*E*). The RDF and the snapshot data indicate that both HCQ and 7CQ prefer to stack with the quartet and loop regions of G4 (Fig. 5, *B*–*E*), in agreement with the NMR results. However, the preference for the accommodation of the HCQ and 7CQ in the loop and quartet region is different as HCQ prefers to stack more with the loop than the quartet of G4, whereas 7CQ interacts with both the loop and quartet of G4. The RDF of the HCQ and 7CQ with the phosphate backbone depict a distinct difference as the charged side chain of HCQ interacts strongly with the phosphate backbone than 7CQ (Fig. 5*C*). The RDF and snapshot (Fig. 5*E*) of the simulation indicate that the strong binding of the charged side group of the HCQ with the phosphate backbone cooperates with the stacking of its quinoline group with the quartets and loops of G4 which probably causes the strong binding of HCQ than 7CQ with G4.

The time-dependent variation of the distance for the HCQ and 7CQ with the guanine of the quartet and thymine of the loop of G4 indicate that binding of the HCQ is stable, whereas 7CQ shows a higher degree of the fluctuation indicating that the interaction of HCQ with quartet and loop of the G4 is more stable than 7CQ (Fig. S15, *A*–*D*). The interaction energy of the drug molecules with G4 depicts that the propensity of the interaction of HCQ with G4 is significantly higher than the 7CQ (Fig. S15*E*), in line with the binding data. It is also apparent that the positively charged HCQ interacts with G4 predominately through electrostatic interaction whereas the contribution of Lennard–Jones (LJ) interaction is higher for neutral 7CQ. The interaction energy of the HCQ and 7CQ with the quartet, loop, and backbone of G4 suggest that the contribution of the LJ is higher for the interaction of the HCQ and 7CQ with the quartet and loop due to the stacking of the quinoline group of both the drug molecules on the quartet and loop regions of G4 (Fig. S15*E*). However, the contribution of the electrostatic interaction of HCQ with the phosphate backbone of G4 is significantly higher than 7CQ (Fig. S15*E*). The MD simulation in corroboration with the NMR data implies that the electrostatic interaction between the side chain of HCQ and the phosphate backbone of the G4 anchor the enhanced stacking of the quinoline group of HCQ with the quartet and loop of G4 leading to the higher binding of HCQ than 7CQ. The effect of the binding of drugs with G4 can influences the interaction energy of the quartets of, hence, the interaction energy of the different quartets of G4 in the absence and presence of HCQ and 7CQ was calculated (Fig. S14). The data suggest that the interaction energy of the quartets is marginally higher on the binding of the G4 with HCQ than 7CQ indicating that the binding of HCQ provides enhanced stability to G4 than 7CQ as observed from the T_m_ data.

## Discussion

Through the integration of *in vitro* and *in vivo* methodologies, we elucidate the impact of FDA-approved drugs, HCQ, CQ, and its derivatives, on gene regulation and tumor progression through the stabilization of G4, particularly to c-myc, and highlight their potential therapeutic implications. The fluorescence binding assays reveal that both HCQ and CQ exhibit a strong affinity for G4 structures associated with oncogenes. Comparative analyses with their analogs, Qn and 7CQ, demonstrate that the charged groups and side chains of HCQ and CQ play a pivotal role in enhancing their binding propensity to G4 structures. Additionally, thermal melting experiments show that HCQ and CQ significantly stabilize the G4 structure of the c-myc oncogene compared to their analogs, indicating their superior G4-stabilizing potential. The molecular level information of the binding of the HCQ with the G4 structure has been speculated using the ^1^H-NMR in the imino region of the G4 and MD simulation. The NMR and MD simulation data suggest that electrostatic interaction between the side chain of HCQ and the phosphate backbone of the G4 anchors the enhanced stacking of the quinoline group of HCQ with the quartet and loop of G4. There are earlier reports in which the ligand has been found to bind with the G4 by stacking at one end of the G4 (44, 49).

Furthermore, our *in vitro* cellular experiments with breast cancer cells demonstrated significant stabilization of DNA G4 structures, as evidenced by the pronounced nuclear foci of BG4 observed following HCQ exposure. RNase treatment following HCQ exposure revealed prominent nuclear BG4 foci, highlighting HCQ's potential selectivity for stabilizing DNA G4 structures over RNA G4 structures. This finding reveals HCQ's potent ability to selectively target and stabilize DNA G4 structures within a cellular context. The analysis of c-myc in breast cancer cells depicts a significant reduction in protein expression following HCQ exposure compared to other oncogenes. HCQ and CQ share a similar structural framework, differing primarily by the presence of a hydroxyl (–OH) group in the side chain of HCQ. Despite this minor difference, HCQ exhibits greater G4 stabilizing ability and enhanced cellular activity than CQ. This suggests that, beyond the electrostatic interactions between the positively charged side chain and the phosphate backbone of G4, the hydrogen-bonding capability of the OH group in HCQ plays a pivotal role by potentially interacting with nucleobases. The structure-activity relationship observed between HCQ and CQ highlights the importance of incorporating hydrogen-bonding functional groups into the charged side chains of aminoquinoline-based drug molecules to enhance their biological efficacy toward DNA targets.

We have also explored whether HCQ can enhance the efficacy of DOX, a standard treatment regimen for breast cancer. The *in vitro* cell-based experiments showed a significant reduction of invasion and metastasis of breast cancer cells when the cells were treated with both HCQ and DOX than the cells treated with DOX alone. Therefore, the study revealed that the combination of HCQ with the frontline chemotherapeutic drug such as DOX augments its antineoplastic activity beyond either single-agent treatment. Furthermore, a preclinical allograft murine model was employed to validate cell-based observation in an *in vivo* setting. The data indicate that higher doses of HCQ as a monotherapy sufficiently reduce tumor growth; however, comparatively lower HCQ doses are only effective in restricting tumor progression when combined with DOX treatment.

The study indicates that HCQ exhibits biological effects at concentrations of 50 to 100 μM. Pharmacokinetic data suggest that these intratumoral levels may be clinically achievable, owing to the favorable tumor-to-plasma partitioning of HCQ (50, 51). Furthermore, clinical data indicate that HCQ is generally well tolerated at doses up to 1200 mg/day (50, 51), which corresponds to approximately 49 mg/kg in humans when normalized to body surface area. In the 4T1 murine model, HCQ monotherapy at 50 mg/kg significantly reduced tumor burden; notably, a lower dose of 10 mg/kg HCQ, when combined with 5 mg/kg DOX, achieved a comparable reduction in tumor growth and c-myc expression. The results suggest that comparable therapeutic outcomes may be achieved at clinically relevant doses, particularly when HCQ is administered in combination with cytotoxic agents or delivered *via* targeted platforms, ultimately improving the therapeutic window and minimizing off-target effects and treatment-associated adverse events. Importantly, the potential incorporation of liposome-mediated targeted drug delivery could further enhance the intratumoral concentration of HCQ within the tumor microenvironment, thereby improving therapeutic efficacy while minimizing systemic exposure. This approach could be further extrapolated in future studies to increase local HCQ accumulation in the tumor microenvironment and should be considered for the development of more effective and targeted therapeutic strategies. Our data showed no measurable difference in DOX uptake upon HCQ treatment, suggesting that the combined effect is largely pharmacodynamic. The observed synergy between HCQ and DOX appears to arise primarily from HCQ-mediated transcriptional repression of c-myc *via* stabilization of G4 DNA structures, however, the role of HCQ on endosomal entrapment or intracellular trafficking (52) cannot be excluded.

Earlier studies have proposed diverse mechanisms for the anticancer activity of HCQ and CQ, though with inconsistent outcomes. These drugs have been suggested to suppress immune function, inhibit autophagy, and induce tumor cell death. For example, HCQ has been reported to potentially diminish the efficacy of anti-PD1 immune checkpoint blockade in tumor immunotherapy. Furthermore, CQ has been proposed to regulate p53 protein and activate p53-dependent transcription of proapoptotic genes, while some studies indicate CQ’s anticancer activity may occur independently of the p53 pathway.

In contrast to these varied findings, our study highlights a distinct mechanism of action demonstrating that quinoline-based drugs particularly HCQ specifically recognize and stabilize the c-myc G4, leading to the downregulation of c-myc mRNA and protein levels. This downregulation correlates with a significant reduction in tumor growth. Importantly, our study also shows that these drugs enhance the efficacy of frontline chemotherapeutic agents, providing a new therapeutic approach for the treatment of breast cancer.

## Conclusion

A variety of synthetic small molecules have been reported to bind to the c-myc G4, however, these molecules could not be used as drug molecules due to the poor biocompatibility and pharmacological issues. In this regard, our work demonstrates the capability of the known FDA-approved immunomodulator drugs HCQ and CQ which are in the preclinical trial stage for the cancer to target specifically the c-myc expression through direct G4 DNA interaction. The recognition of c-myc-G4 with the drug is mainly triggered by the electrostatic interaction between the charged side chain of the HCQ with the phosphate backbone of G4 which anchors the stacking of the quinoline ring of HCQ on the quartet and loop of G4. Beyond intrinsic anticancer effects, this study also suggests that HCQ may resensitize cells to chemotherapy that would ordinarily upregulate prosurvival c-myc levels. Our data advocate further preclinical development of this approved drug for potential clinical benefit in breast cancer care.

## Experimental procedures

### Chemicals

HPLC grade pure DNA sequences of c-myc, bcl2, c-kit, KRAS, VEGF, and duplex DNA were purchased from Sigma-Aldrich (The detail of sequences are provided in Table 1). HCQ, CQ, quinine (Qn, Fig. 1*A*), 4-amino-7-CQ (Fig. 1*A*), and KCl were procured from Sigma-Aldrich. The purity of all the chemicals is ≥ 95% by HPLC analysis. All the DNA samples were dissolved in 10 mM Tris–HCl buffer (pH = 7.4) and annealed in the 2 mM KCl salt by increasing the temperature up to 90 °C, followed by cooling at room temperature.

**Table 1:** The name and detail of the sequences used in this study

Name	Sequences
c-myc	TGAGGGTGGGTAGGGTGGGTAA
bcl2	AGGGGCGGGCGCGGGAGGAAGGGGGCGGA
c-kit	CGGGCGGGCGCGAGGGAGGGG
KRAS	GGGAGGGAGGGAAGGAGGGAGGGAGGGA
VEGF	CCCGGGGCGGGCCGGGGGCGGGGTCCCGGCGGGGCGGAG
Duplex	CAATCGGATCGAATTCGATCCGATTG

### Fluorescence measurements

The emission spectra of the immunomodulator drugs dissolved in Tris–HCl buffer (pH = 7.4) are measured by exciting at the isosbestic point of the absorption of DNA and HCQ (295 nm) in the absence and presence of different DNA sequences using Fluoromax-4 (Horiba Scientific) instruments at 25 °C. The absorption spectra of DNA and HCQ exhibit a common intersection at 295 nm, corresponding to an isosbestic point (Fig. S1). The presence of this point indicates that both DNA and HCQ share the same absorbance at 295 nm. Therefore, 295 nm was selected as the excitation wavelength for emission measurements, as it accurately reflects the excitation of the drug–DNA complex. The slit width of the measurement is fixed at 2 nm. All the fluorescence spectra measured in this study have been baseline-corrected by subtracting the buffer contribution. The binding constant of drug molecules (10 μM) with different sequences of DNA has been estimated by measuring the fluorescence of these drugs with increasing concentrations of different DNA sequences (∼up to 3 μM) until the decrease in the fluorescence signal is constant. The binding constant was calculated using the modified Stern–Volmer equation shown in equation Equation 1.

(1) $$\log\left(\frac{F_{0}-F}{F}\right)=\log\left(K_{b}\right)+\text{nlog}\left[\text{DNA}\right]$$

Here, F_0_ and F correspond to the fluorescence intensity of the drug in the absence and presence of DNA. K_b_ and n represent the binding constant and stoichiometry of the binding of drugs with different sequences of DNA. The binding free energy ΔG_b_ (kcal/mole) of drugs with DNA sequences was calculated at 25 °C using Equation 2.

(2) $${\Delta G}_{b}=-\text{RT}\;\ln\left(K_{b}\right)$$

### CD and T_m_ measurements

The CD spectra of the G4 structure of each sequence of DNA in the presence of drugs in the range of 220 to 310 nm were measured using JASCO (J1500) spectrometer to understand the effect of the drugs on the G4 structure of the different sequences of DNA. The drug-induced stabilization in the G4 structure of the different sequences (5 μM) was estimated by measuring the T_m_ of the G4 structure of DNA without and with drug molecules (50 μM) in 2 mM of KCl. The T_m_ of each G4 structure was estimated by measuring the temperature-dependent change in the ellipticity of the characteristic peak of the G4 structure (∼260 nm). The temperature of the system was varied from 10 °C to 90 °C in the interval of 3 °C. Each measurement is an average of three different CD spectra.

### Competitive FRET-melting assay

Fluorescein amidites (6-FAM attached as donor at the 5′end and carboxy TAMRA attached as acceptor at the 3′ end of c-myc has been used for the competitive FRET-melting assay. The competitor G4 or duplex DNA sequences have been mixed in a different molar ratio (1:1 and 1:10) with the labeled c-myc to understand the competitiveness between c-myc G4 with other G4s/duplex DNA sequences. The 2 μM of HCQ has been added as the ligand. The FRET measurement has been performed using the FluoroMax-4 (Horiba Scientific) instruments at 25 °C. The fluorescence spectra of both FAM (∼520 nm, donor) and TAMRA (∼580 nm, acceptor) attached with c-myc (∼25 nM) excited at the peak maxima of FAM (λ_ex_ = 492 nm) have been measured in buffer and HCQ (2 μM) by varying the temperature of the cuvette through the peltier attached with a fluorometer. The temperature was varied in the range of 10 °C to 95 °C in the interval of 5 °C. Each fluorescence spectrum is an average of three different measurements. Further, the fluorescence measurement of both the donor and acceptor attached with c-myc with the HCQ has been performed in the presence of different G4s and duplex. The FRET efficiency (E) has been estimated using the Equation 3.

(3) $$E=F_{A}/\left(F_{D}+F_{A}\right)$$

F_D_ and F_A_ are the fluorescence intensities of the donor (FAM) and acceptor (TAMRA) channels. The melting curve of each measurement has been obtained by plotting the estimated FRET efficiency against the temperature.

### Cell culture

The human cancer cell lines MDA-MB-231 (human TNBC cell line), HeLa (human cervical cancer cell line), murine breast cancer cell line 4T1, and noncancer cell line HEK293 were acquired from the American Type Culture Collection and cultured in Dulbecco's modified Eagle's medium (DMEM, Gibco) supplemented with 10% FBS and 100 U/ml penicillin-streptomycin (Invitrogen).

### BG4 immunofluorescence

Cells were grown on glass coverslips without and with exposure to drugs (HCQ, CQ, and 7CQ) and then fixed for further process. Fixed cells were permeabilized with 0.2% Triton X-100 and then were blocked with 3% bovine serum albumin (BSA). After blocking, before staining with BG4, cells were treated with 20 μg/ml of DNase and RNase for 1 h at 37 °C min. Cells were incubated with a commercial BG4 antibody and anti-Flag for 1 h and then incubated with anti-rabbit Alexa Fluor 488–conjugated secondary antibodies for 2 h. Samples were costained with 0.1 μM 4′,6-diamidino-2-phenylindole (DAPI) for 10 min and visualized using a laser scanning confocal microscope.

### G-quadruplex ChIP-qPCR

Cells were seeded in 35-mm dishes, and after reaching confluency, they were treated with 50 μM HCQ. Cells were crosslinked with formaldehyde, and excess formaldehyde was quenched using 1 M glycine. Nuclei were extracted using a nuclear extraction buffer, and sonication was performed to shear the DNA to an average size of 300 bp. Protein A/G magnetic beads were prepared and incubated with BG4 and Flag antibodies for 4 to 6 h. The chromatin sample prepared earlier was incubated with the antibody-conjugated beads overnight at 4 °C with gentle rotation. On the following day, the beads were collected using a magnet, and the supernatant was discarded. The bead-bound chromatin was treated with RNase and proteinase K, followed by purification of the enriched immunoprecipitated DNA. The immunoprecipitated sample (IP and Mock) and the input were used to quantify G4 enrichment *via* quantitative PCR, using Fast SYBR PCR mix (Applied Biosystems), with ABI7500 quantitative PCR machine. Cycling conditions were 95 °C for 20 s followed by 50 cycles of 3 s at 95 °C and 30 s at 53 °C. We employed primer pairs that target G4-ChIPpositive and G4-ChIPnegative regions (Table S1). Relative enrichments were derived with respect to their IgG control.

### ChIP-qPCR for SP1 enrichment

Cells were seeded in 35-mm dishes, and after reaching confluency, they were treated with 50 μM HCQ for the indicated duration. Cells were crosslinked with formaldehyde, and excess formaldehyde was quenched using 1 M glycine. Nuclei were extracted using a nuclear extraction buffer, and chromatin was sonicated to achieve an average DNA fragment size of ∼300 bp. Protein A/G magnetic beads were prepared and incubated with SP1 antibody for 4 to 6 h at 4 °C. The prepared chromatin samples were then incubated overnight at 4 °C with the antibody-conjugated beads under gentle rotation. The following day, beads were collected using a magnetic stand, and the supernatant was discarded. The bead-bound chromatin was treated sequentially with RNase and proteinase K, followed by purification of the immunoprecipitated DNA. Both the immunoprecipitated samples (IP and mock) and the input DNA were subjected to quantitative PCR analysis using Fast SYBR PCR mix (Applied Biosystems) on an ABI7500 quantitative PCR system. Cycling conditions were 95 °C for 20 s followed by 50 cycles of 3 s at 95 °C and 30 s at 53 °C. Primer pairs specific for the c-myc and KRAS promoter regions were used for quantification. Relative enrichment of SP1 at these loci was calculated with respect to the IgG control.

### Western blotting

To find out the expression of c-myc and bcl2 in cancer cells, 5 × 10^5^ cells were seeded per well in a 6-well plate. Cells were kept overnight in complete growth media to adhere. They were then treated either with different concentrations of HCQ (100 μM, 50 μM, 25 μM, and 12.5 μM) or with 50 μM CQ, Qn, 7CQ, and HCQ (dissolved in pH 7 buffer) for 48 h. Cells were then lysed using Laemmli buffer. The lysate was heated at 95 °C for 5 min. SDS-PAGE was performed to separate the proteins and transfer them onto the PVDF membrane, following blocking in 5% BSA in TBS buffer. The membrane was incubated overnight at 4 °C with primary antibodies for specific proteins in 3% BSA in TBS. A secondary antibody was added and incubated for an hour after washing with TBS, 1% Tween-20 three times. Then, blots were developed using ECL. Densitometric analysis was done using ImageJ, and graphs were made using GraphPad Prism 8.

### Flow cytometry

To determine the expression of c-myc, cells treated with 50 μM of HCQ, CQ, Qn, 7CQ, (dissolved in pH 7 buffer) for 48 h were washed with PBS. Then the cells were permeabilized with 1% Triton X-100. Next, cells were incubated with c-myc primary antibody for 1 h, followed by 45 min incubation with Alexa 488–conjugated corresponding secondary antibody. Rabbit IgG was used as isotype control. Cells were then subjected to flow cytometry analysis (BD FACS AriaIII). Acquired data were analyzed using FlowJo (version 8).

### Quantitative real-time PCR

To evaluate the influence of HCQ on the mRNA expression of c-myc, bcl2, c-kit, KRAS, VEGF, and HK-2 in cancer cells (Table S2), cells were seeded at a density of 5 × 10^6^ cells per 35-mm dish. Next cells were treated with different doses (100 μM, 50 μM, 25 μM, and 12.5 μM) of HCQ or fixed 50 μM of HCQ as per desired experiments. Subsequently, total cellular RNA was extracted using the TRIZOL reagent (Life Technologies). cDNA was then prepared from extracted total RNA using the Super-Script III First-Strand cDNA Synthesis System from Invitrogen, following the manufacturer's guidelines. The resulting cDNA was utilized for quantitative real-time PCR, employing the SYBR Green Master Mix (Applied Biosystems), performed on the Step One plus Real-Time PCR System. Data normalization was accomplished using GAPDH as the reference gene, and each experimental sample underwent triplicate analyses for precision. Relative expression levels were computed using the 2^−ΔΔCt^ method. Primer sequences are provided in the Table S2.

### Immunofluorescence

Cells were seeded onto hydrofluoric acid–etched coverslips (35%). Subsequently, cells received treatment with either 50 μM or 100 μM of HCQ. Fixation was carried out using 4% paraformaldehyde for 30 min, followed by a 10-min permeabilization step using 0.1% Triton X-100. After three washes with PBS, cells were blocked with 5% BSA for 1 h. Overnight incubation with c-myc antibody followed, and cells were then subjected to a 1-h treatment with Alexa Fluor 488–conjugated rabbit secondary antibody postwashing. Slides were exposed to DAPI for 10 to 20 min, followed by additional PBS washing. Finally, cell imaging was performed using a Carl Zeiss laser scanning confocal microscope. For the DOX uptake experiment, MDA-MB-231 breast cancer cells were seeded onto hydrofluoric acid-etched coverslips and treated with or without HCQ (50 μM) for the indicated duration. Subsequently, cells were incubated with DOX (1.5 μM) under standard culture conditions. Following treatment, nuclei were counterstained with DAPI. Fluorescence images were acquired using a fluorescence microscope, with DAPI signal representing nuclear staining (blue) and DOX uptake visualized as red fluorescence. Quantification of DOX fluorescence intensity was performed using ImageJ software, and data were expressed as relative fluorescence intensity.

### Cell viability assay

To assess cell viability, MDA-MB-231 or HEK 293 cells were seeded at a density of 8 × 10^3^ cells per well in 96-well plates. After overnight incubation in complete growth media (DMEM), the cells were treated with the different concentrations of HCQ, CQ, and 7CQ. In another experiment for understanding the effect of known anticancer drug DOX with HCQ, the cells were treated with 50 μM of HCQ, 1.5 μM of DOX, or a combination of both compounds. These treatments were administered for 72 h after which MTT reagent was added. Following a suitable incubation period, the formazan crystals that developed were dissolved in DMSO, and the absorbance was measured at 570 nm to determine cell viability. The resulting data were analyzed, and cell viability graphs were generated using GraphPad Prism 8.

### Migration assay

MDA-MB-231 cells were seeded onto 35-mm dishes, permitting them to attain 80 to 85% confluence. Subsequently, the cells underwent treatment with HCQ (50 μM), DOX (1.5 μM), or a combination of both. On the same day, uniform scratch lines were created using a micro tip. After a 48-h interval, images were captured to assess migratory changes. The migratory capacity of cells was quantified by contrasting them with the baseline 0-h treatment.

### Invasion assay

Cells were equally seeded onto Matrigel-coated Transwell chambers. After treatment with HCQ (50 μM), DOX (1.5 μM), or a combination, the upper chamber contained a serum-free medium, while FBS-containing DMEM was in the lower chamber. Following 48 h of incubation, cells that invaded the lower membrane surface were stained with crystal violet solution. After removing upper surface cells, the total invaded cells were counted in ten randomly chosen fields using a bright-field microscope.

### Animal studies

Balb/c mice were purchased from W.B. Livestock Dev. Corpn. Ltd. All the experiments were conducted in accordance with the institutional guidelines and approved by the Institutional Animal Ethical Committee, approval number IACS/Institutional Animal Ethical Committee/2021-09, dated 21/12/2021. Six- to eight-week old BALB/c mice were subcutaneously injected (1 × 10^6^ 4T1 cells resuspended in PBS buffer) in the lateral flank. Mice (n = 5 per group) were then divided into groups and were then subjected to control (saline), oral gavage of either 10 mg/kgbdwt or 50 mg/kgbdwt HCQ, 5 mg/kg of DOX treatment *via* tail vein injection, or a combination of both HCQ and DOX treatment were carried on the 1-day interval from the fifth day until the day of sacrifice. Tumor size was measured post sacrifice at day 25 post tumor cell injection. Tumors were removed weighed and compared between the groups. The tumor tissue was homogenized in a lysis buffer to extract proteins. Western blot analysis was then performed to measure the c-myc levels in different tumor groups.

### NMR measurement

The ^1^H-NMR measurements in the imino region of c-myc (100 μM) annealed in 2 mM KCl were measured in the presence of HCQ and 7CQ in H_2_O/D_2_O 90%/10% mixture to understand the binding modes of HCQ with c-myc. The NMR measurements were performed using a 600 MHz spectrometer at 25 °C. Each spectrum is an average of 10,000 scans.

### MD simulation

MD simulation was conducted utilizing the parallel G4 structure of c-myc (PDB: 1AXV) (53), as described by the Amber99 force field with OL15 correction (54). Three distinct systems were prepared: c-myc without a drug, c-myc with HCQ, and c-myc with 7CQ. The parameters for the protonated form of HCQ (55) and the neutral structure of 7CQ were adopted from the Generalized Amber Force Field using the Antechamber package (56). The solvation of the systems was achieved using TIP3P water molecules (57). For the first system, the G4 structure was solvated in a cubic box with sides measuring 6 nm, maintaining a 0.1 M salt concentration without a drug molecule. Subsequently, for the second system, six HCQ cations and six SO_4_^2-^ anions were introduced. For the third system, six 7CQ molecules were randomly inserted within the box. Ions (K^+^, Cl^-^) were employed to maintain a 0.1 M bulk concentration of salt and neutralize the negative charge of G4. The simulations were conducted using GROMACS-2016.3 software (58). Initially, all systems underwent energy minimization for 5000 steps using the steepest descent method, followed by position-restrained equilibration for 200 ps. Subsequently, NVT (300 K) and NPT (1 atm) simulations were performed for 1 ns each to properly equilibrate the solvent and ions. The production MD run spanned 1 μs for each system, employing a time step of 2 fs. The LINCS algorithm was utilized to constrain bond lengths between heavy atoms and hydrogen (59). Temperature and pressure were regulated through the velocity rescaling (V-rescale) thermostat (60) (τ = 0.1 ps) and Parrinello-Rahman pressure coupling (61) (τ = 2 ps), respectively. The cut-off radius for neighbor searching and nonbonded interactions was set at 1 nm, with the nonbonded pair list updated every ten steps. The particle Mesh-Ewald method with a grid spacing of 0.12 nm and fourth-order interpolation was applied to account for long-range electrostatic interactions (62). Trajectory analysis utilized GROMACS tools, and snapshots were generated using Visual Molecular Dynamics software (VMD 1.9.3) (63). The RDF of the HCQ and 7CQ with different parts of G4 was calculated to get an idea of the interaction of these molecules with G4. The following atoms of the G4 have been considered to calculate the RDF of the quartet, loop, and the phosphate backbone of the G4 with drug molecules: 1) electronegative atoms of the guanine bases for the quartet region, 2) the electronegative atoms of the T and A bases for the loop regions, and 3) phosphorus of the phosphate backbone have been selected for RDF calculations. The nitrogen of the quinoline ring of HCQ or 7CQ (denoted as N2) has been considered for the RDF calculation of the ring part of drug molecules, whereas the side chain has been represented by the O and the N atoms of the sidechain for HCQ and 7CQ, respectively. The total interaction energy of the drug with quartet, loop, and phosphate backbone of G4 and its coulombic and LJ terms has been calculated to get the idea of the energetics of binding of these drug molecules with G4. For the energy calculation, quartet: heavy atoms of the G bases of the rings without phosphate backbone; loop: heavy atoms of the A and T bases without phosphate backbone; and phosphate: heavy atoms in the phosphate regions were selected.

## Data availability

Data will be made available on request.

## Supporting information

Spectroscopy data of the binding and melting of different G4 sequences with drug molecules NMR data of the binding of c-myc G4 with 7CQ, the MD simulation data of the G4 structure of c-myc with HCQ and 7CQ, and quantification of the *in vitro and in vivo* biological experiments. This article contains Supporting information.

## Conflicts of interest

The authors declare that they have no conflicts of interest with the contents of the article.
