# Supplementary material for: G-Quadruplex–specific action of chloroquine-based immunomodulator drugs to inhibit the cancer progression
Source: J Biol Chem. 2025 Sep 22;301(11):110753. doi: 10.1016/j.jbc.2025.110753 (PMC12554177; doi:10.1016/j.jbc.2025.110753)
Supplement: Supporting information [file mmc1.docx]

**Supporting Information**

**G-Quadruplex Specific Action of Chloroquine based Immunomodulator Drugs to Inhibit the Cancer Progression**

Sunipa Sarkar^a^, Akash Chatterjee^b^, Subhojit Paul^b^, Asim Bisoi^a^, Prosenjit Sen^b*^, Prashant Chandra Singh^a*^

^a^School of Chemical Sciences, Indian Association for the Cultivation of Science, Jadavpur, Kolkata 700032

^b^School of Biological Sciences, Indian Association for the Cultivation of Science, Jadavpur, Kolkata 700032

*bcps@iacs.res.in, [sppcs@iacs.res.in](mailto:sppcs@iacs.res.in)

**Corresponding Author:**

Prof. Prashant Chandra Singh

Indian Association for the Cultivation of Science, Jadavpur, Kolkata 700032

E-mail: [sppcs@iacs.res.in](mailto:sppcs@iacs.res.in)


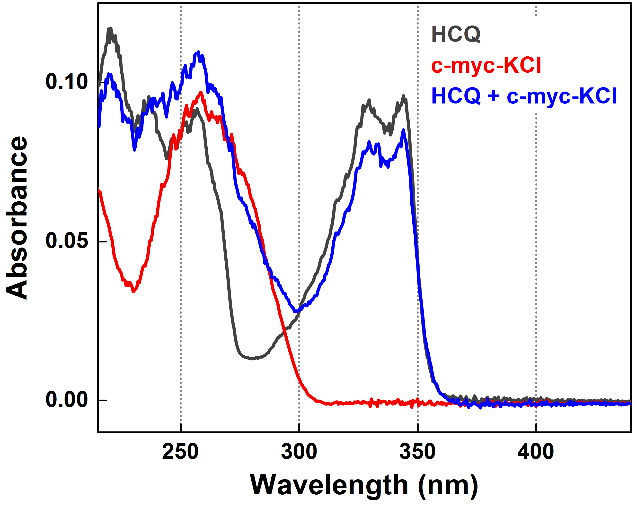


**Figure S1:** The absorption spectra of c-myc DNA (3 µM), HCQ (10 µM) and DNA with HCQ. The isosbestic point of DNA and HCQ is ~295nm.

**
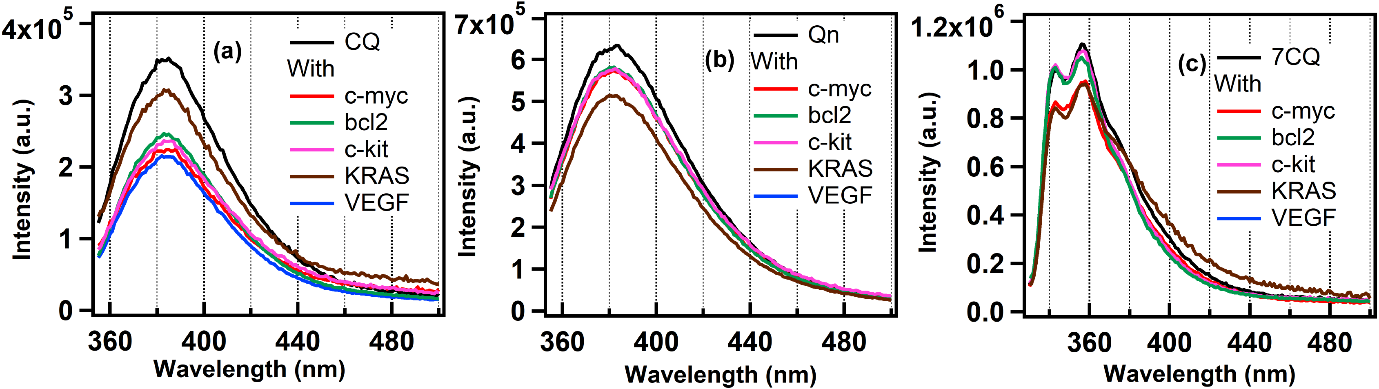
**

**Figure S2:** The emission spectra of CQ (10 µM, a), Qn (10 µM, b), 7CQ (10 µM, c) in buffer and different G4 DNA (3 µM) sequences.


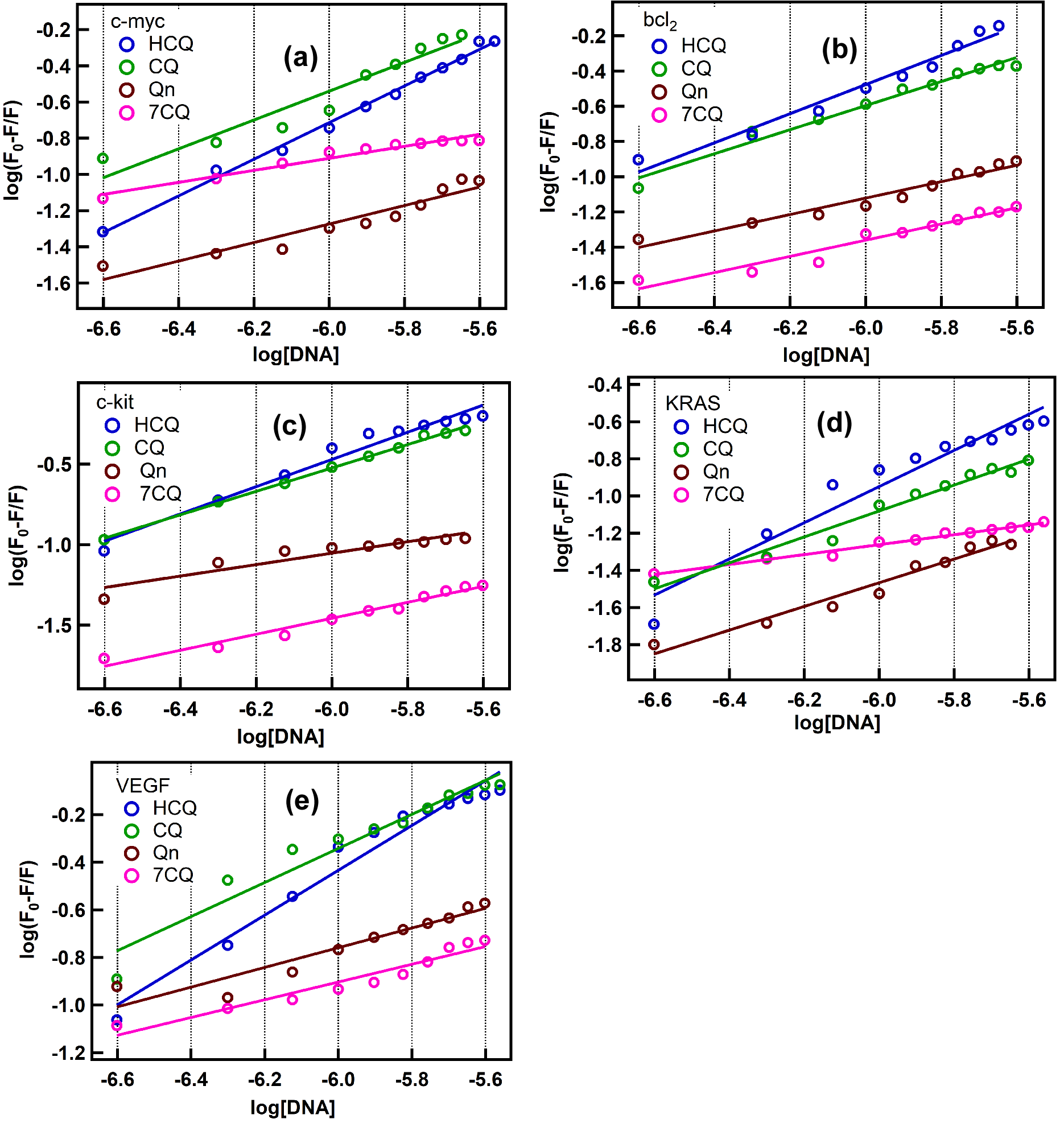


**Figure S3:** The plot of the change in fluorescence intensity of HCQ, CQ, and its analogs Qn and 7CQ with respect to the different concentrations of c-myc(a), bcl2 (b), c-kit (c), KRAS (d), and VEFG (e) DNA sequences, respectively. The data has been fitted with the modified Stern-Volmer equation to calculate the binding constant value of the drug with G4 sequences. The concentration of drugs used for the binding measurements is 10 μM.


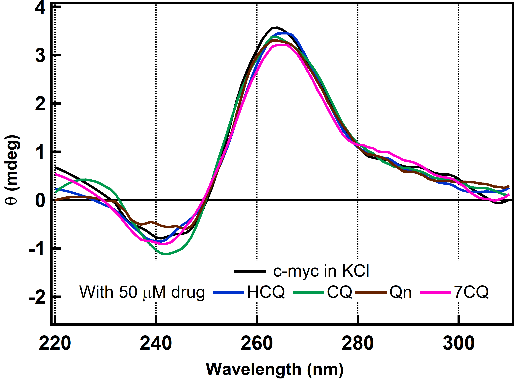


**Figure S4:** The CD spectra of c-myc (5 μM) in KCl salt and 50µM of HCQ, CQ, Qn, and 7CQ drugs.


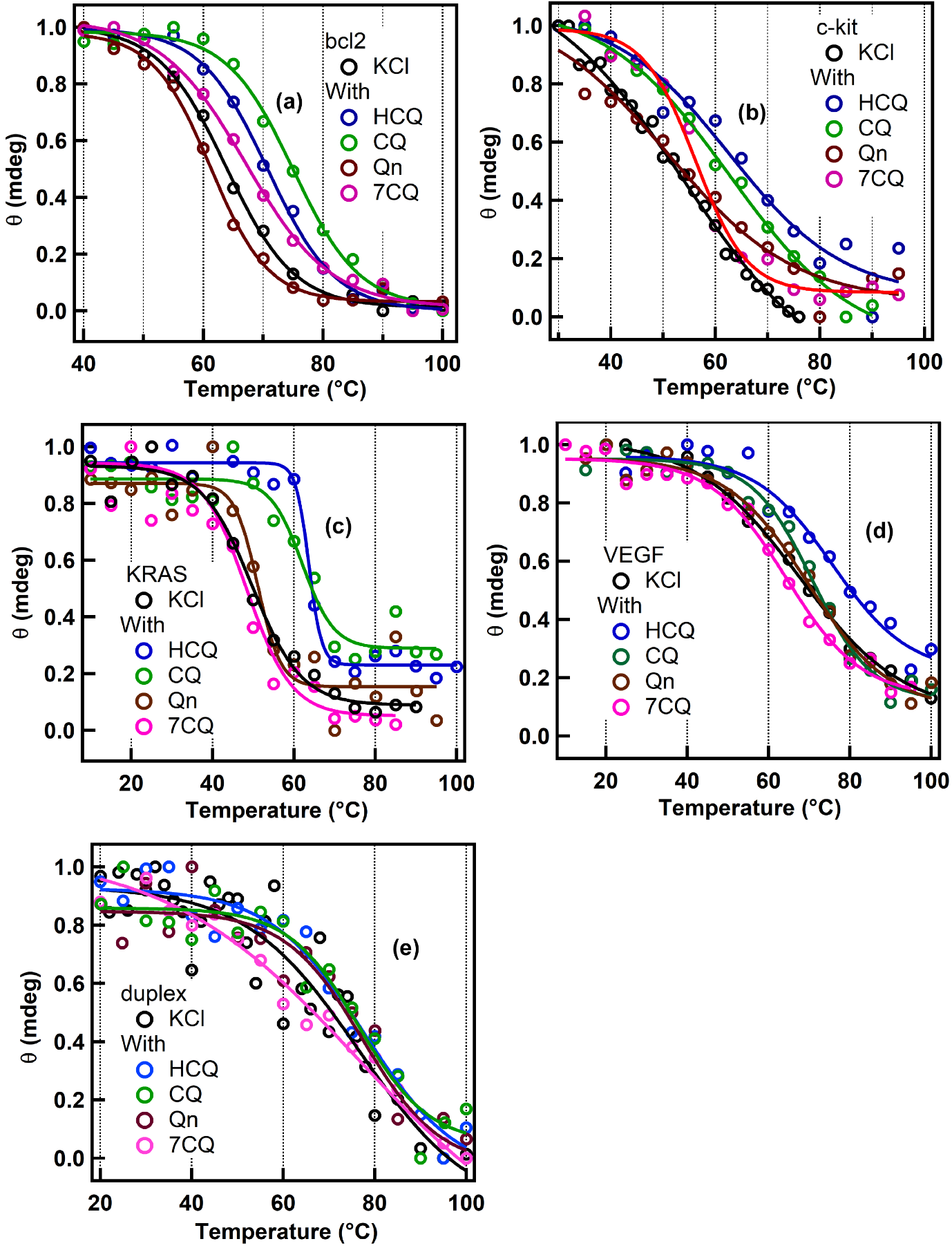


**Figure S5:** The melting curves of bcl2 (5µM, a), c-kit (5 µM, b), KRAS (5 µM, c), VEGF (5 µM, d), duplex (5µM, e) in KCl and the presence of different drug molecules (50 µM each).

**
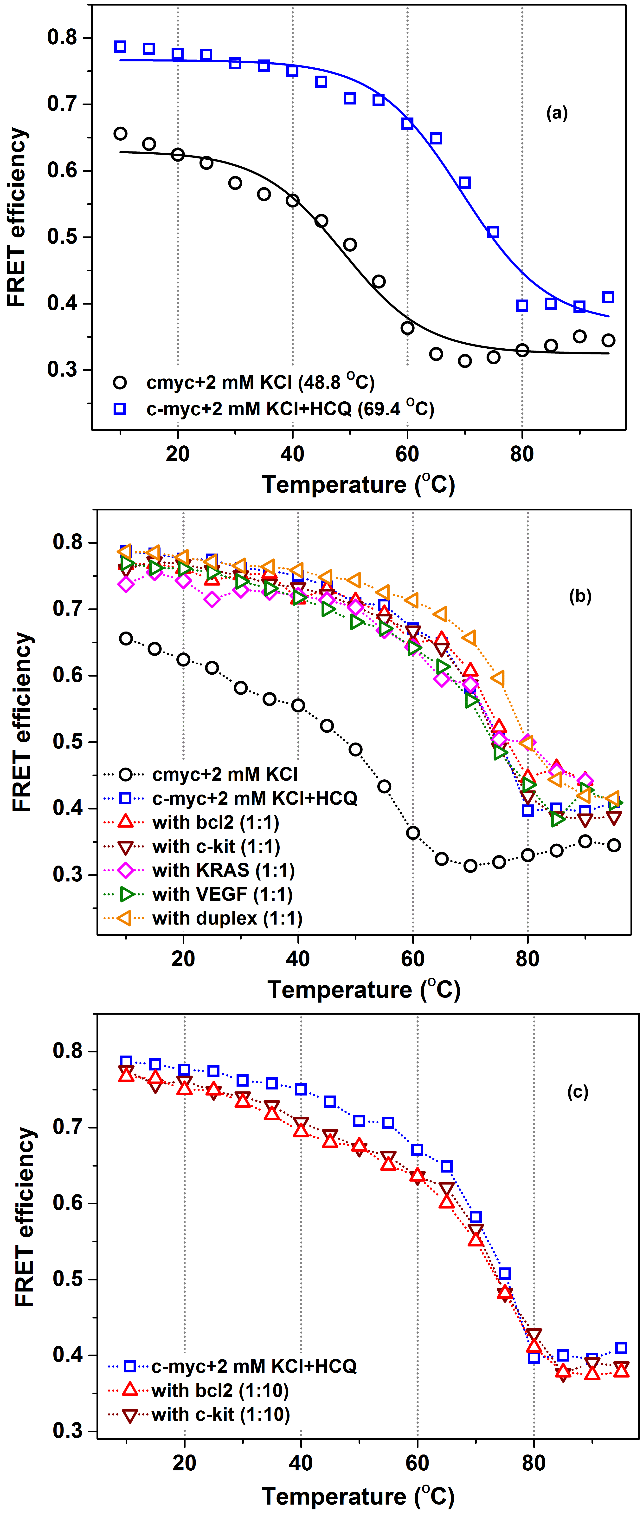
**

**Figure S6:** (a) FRET efficiency (F_A_/ F_A_ + F_D_) of labeled c-myc G4 (25 nm) against increasing temperature in KCl (2 mM) and HCQ (2 µM). FRET efficiency of labeled c-myc bound with HCQ on adding other G4 and duplex DNA strands in different molar ratios (1:1 in b and 1:10 in c). F_D_ and F_A_ refer to the fluorescence intensity of the donor (6-FAM) and acceptor (TAMRA) channels attached with c-myc respectively.


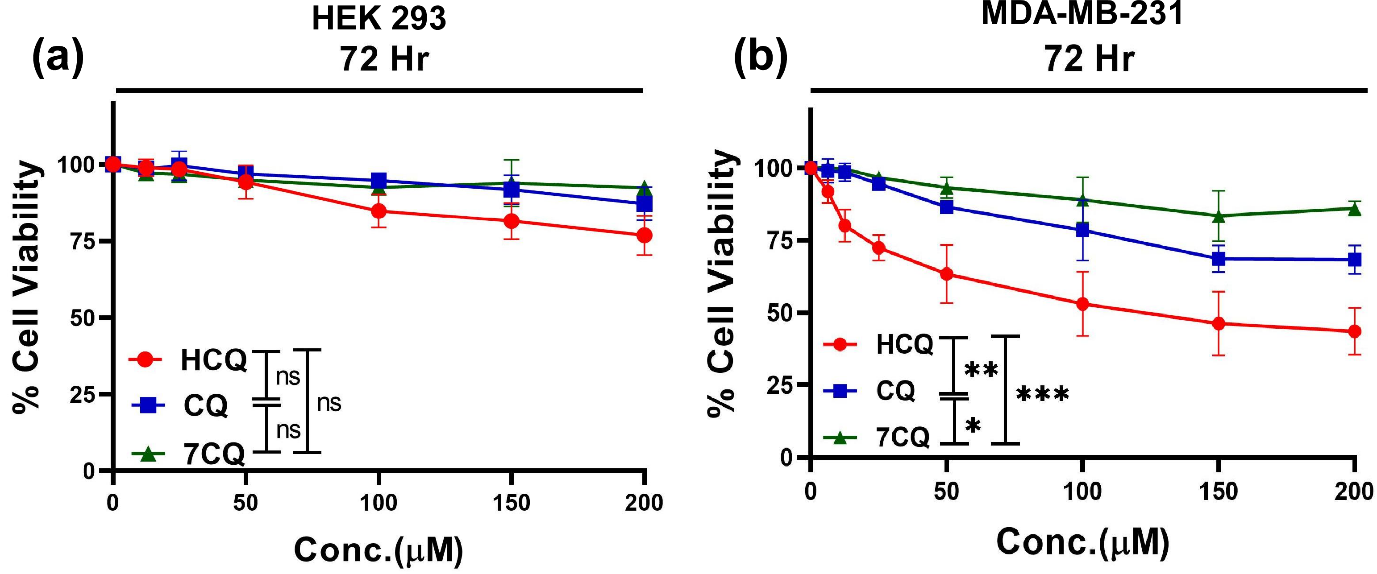


**Figure S7.** Cell viability of HEK293 cells (a), a non-cancerous human embryonic kidney-derived cell line, and MDA-MB-231 cells (b), a model of triple-negative breast cancer after 72 hrs treatment of different concentrations of HCQ, CQ and 7CQ. The error bars indicate the mean ±SD of three independent experiments. The statistical significance levels denoted as follows: ns = not significant, P>0.05, *P<0.05, ** P<0.01, ***P<0.001, assessed using ANOVA with Tukey post hoc test for multiple comparisons.


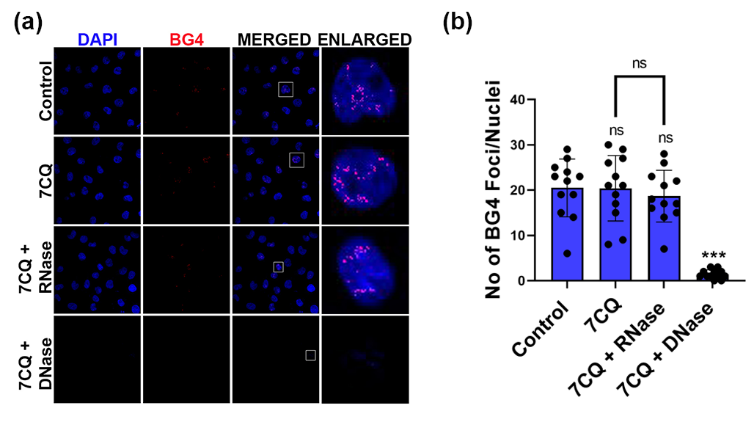


**Figure S8:** Immunofluorescence images stained for nucleus (DAPI), G4 structures showing BG4 foci (red), and merged images in the absence (control) and the presence of 7CQ alone as well as along with RNase and DNase, respectively using MDA-MB-231 breast cancer cell line. (b) Quantification of BG4 stained foci was performed using Image J and represented as bar graph. The error bars indicate the mean ±SD of three independent experiments and the statistical significance levels denoted as follows: ns = not significant, P>0.05, *P<0.05, ** P<0.01, ***P<0.001, ****P<0.0001, assessed using ANOVA with Tukey post hoc test for multiple comparisons.

**Figure S9:** ChIP-qPCR data showing relative enrichment of SP1 at the c-myc and KRAS gene in the vehicle and HCQ-treated cells. The error bars indicate the mean ±SD of three independent experiments and the statistical significance levels denoted as follows: ns = not significant, P>0.05, *P<0.05, ** P<0.01, ***P<0.001, ****P<0.0001.

**
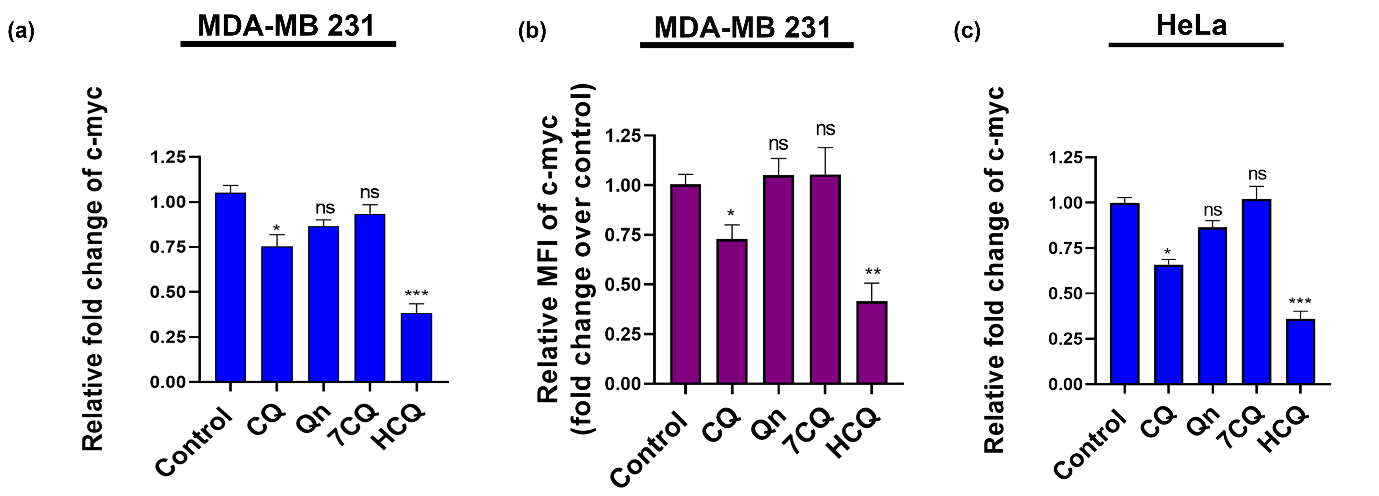
Figure S10:** (a) Densitometric analysis of Western blot of c-myc expression in MDA-MB-231 cell line after treatment with CQ, Qn, 7CQ, and HCQ (dissolved in pH 7 buffer) for 48 hrs using ImageJ. (b) Quantification of the relative MFIs obtained from the flow cytometry analysis of c-myc expression after drug treatments. (c) Densitometric analysis of Western blot of c-myc expression in HeLa cell line after treatment with CQ, Qn, 7CQ, and HCQ (dissolved in pH 7 buffer) for 48 hrs using ImageJ. All the graphs were generated using GraphPad Prism8, presenting the combined (mean) outcomes derived from three independent experiments. The error bars indicate the range of variability observed across these repetitions (mean ± standard deviation, n=3), with statistical significance levels denoted as follows: ns = not significant, P>0.05, *P<0.05, ** P<0.01, ***P<0.001, ****P<0.0001, assessed using ANOVA with Tukey post hoc test.


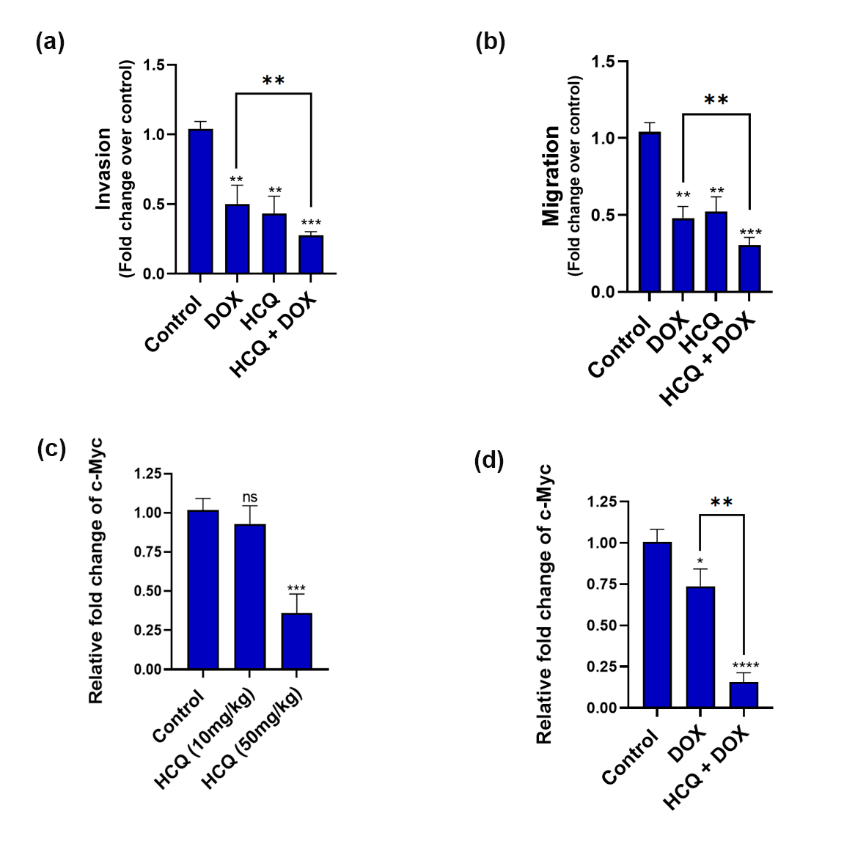


**Figure S11:** (a) Quantification of Transwell invasion assay in MDA-MB-231 cells after the indicated treatments. (b) Quantification of wound healing assay showing the extent of migration of MDA-MB-231 cells after the indicated treatments. (c) Densitometric analysis of Western blot of c-myc expression in tumors from BALB/c mice following the sacrifice. GAPDH was used as a loading control. (d) Densitometric analysis of Western blot data of c-myc expression in tumors following indicated treatments. The error bars indicate the mean ±SD of three independent experiments and the statistical significance levels denoted as follows: ns = not significant, P>0.05, *P<0.05, ** P<0.01, ***P<0.001, ****P<0.0001, assessed using ANOVA with Tukey post hoc test for multiple comparisons.


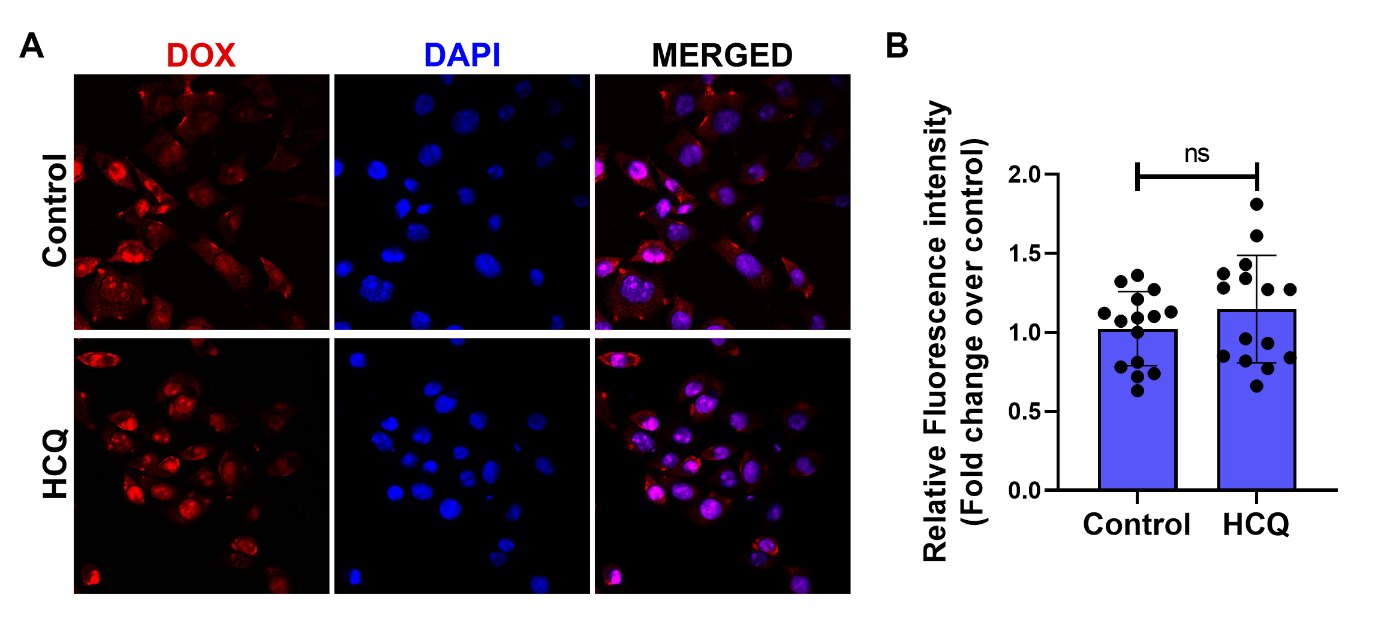


**Figure S12:** (A) Fluorescence microscopy images stained for nucleus (DAPI), Dox uptake (red), and merged images in the absence (control) and the presence of HCQ using MDA-MB-231 breast cancer cell line. (B) Quantification of relative fluorescence intensity of DOX uptake using ImageJ. The error bars indicate the mean ±SD and the statistical significance levels denoted as follows: ns = not significant, P>0.05, *P<0.05, ** P<0.01, ***P<0.001, ****P<0.0001.


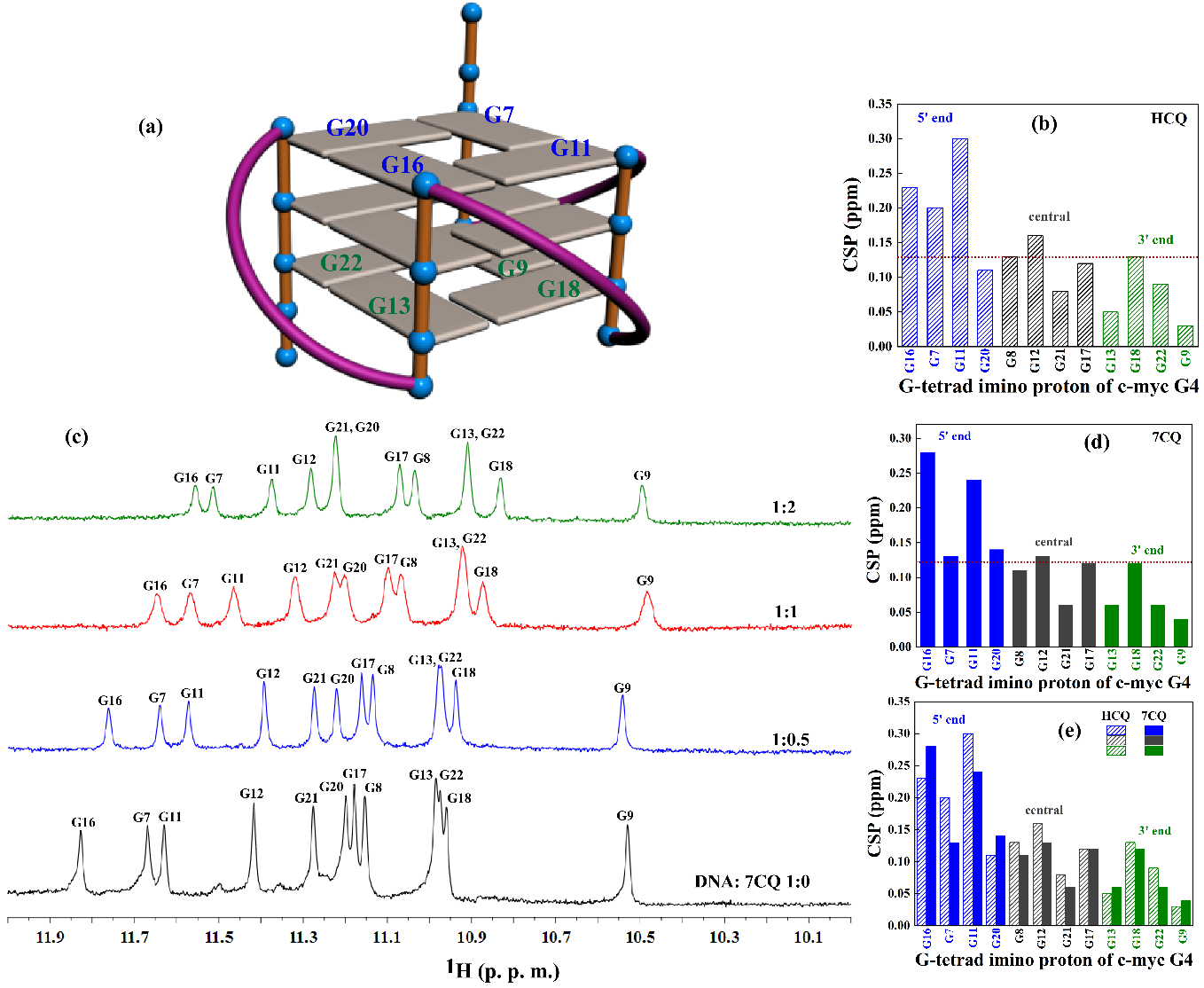


**Figure S13:** (a)The representation of the parallel structure of c-myc. The G4- tetrad at 5’ and 3’ terminals are marked in blue and green colors. (b) The plot of chemical shift perturbation (CSP) for (CSP) each imino protons of c-myc G4-tetrads at 5’end (blue), central (black), and 3’ end (green) terminals in the presence of HCQ. The CSP has been calculated by the subtraction of the position of each imino proton in the case of a 1:2 c-myc-HCQ complex with respect to the only c-myc case. The brown dashed line in Figure b indicates the one standard deviation above the average value of the CSP of all the cases. (c) ^1^H-NMR spectra representing the imino region of the G4 during the titration of c-myc with 7CQ. The G4-imino protons of tetrad and the ratio of c-myc and HCQ are labeled in each spectrum. (b) The plot of chemical shift perturbation for (CSP) each imino protons of c-myc G4 tetrads at 5’ (blue end), central (black), and 3’ (green) terminals in the presence of 7CQ. (c) The comparison of the change of CSP values of c-myc G4 in the case of HCQ and 7CQ, respectively.


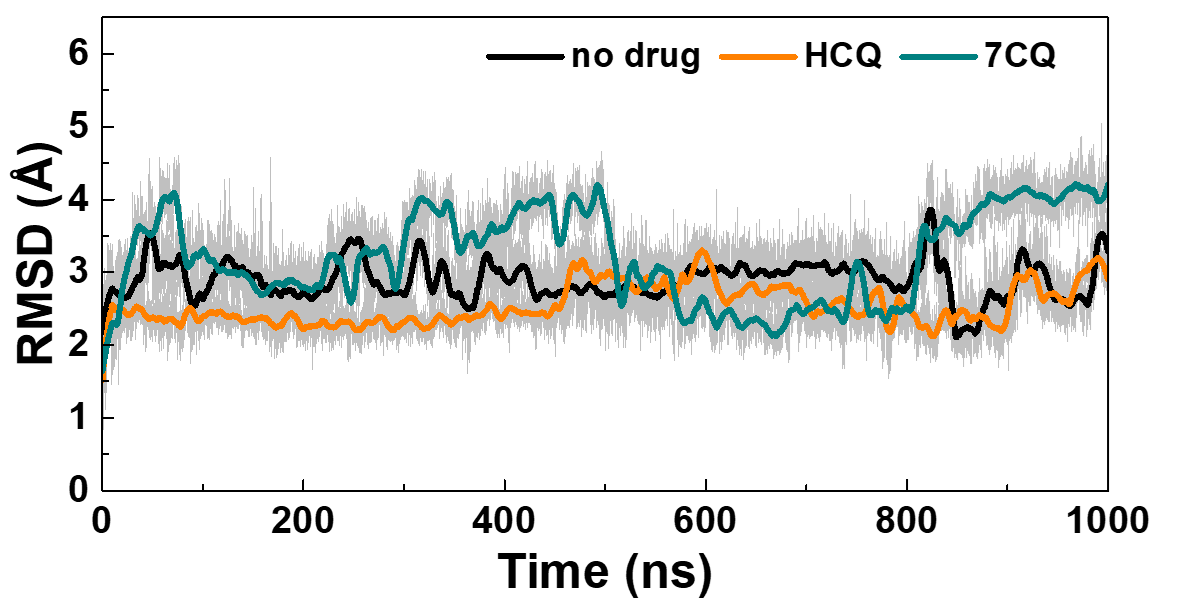


**Figure S14.** The root mean square deviation (RMSD) of G4 structure in the absence of drug (no drug) and in the presence of HCQ and 7CQ for the whole simulation time.


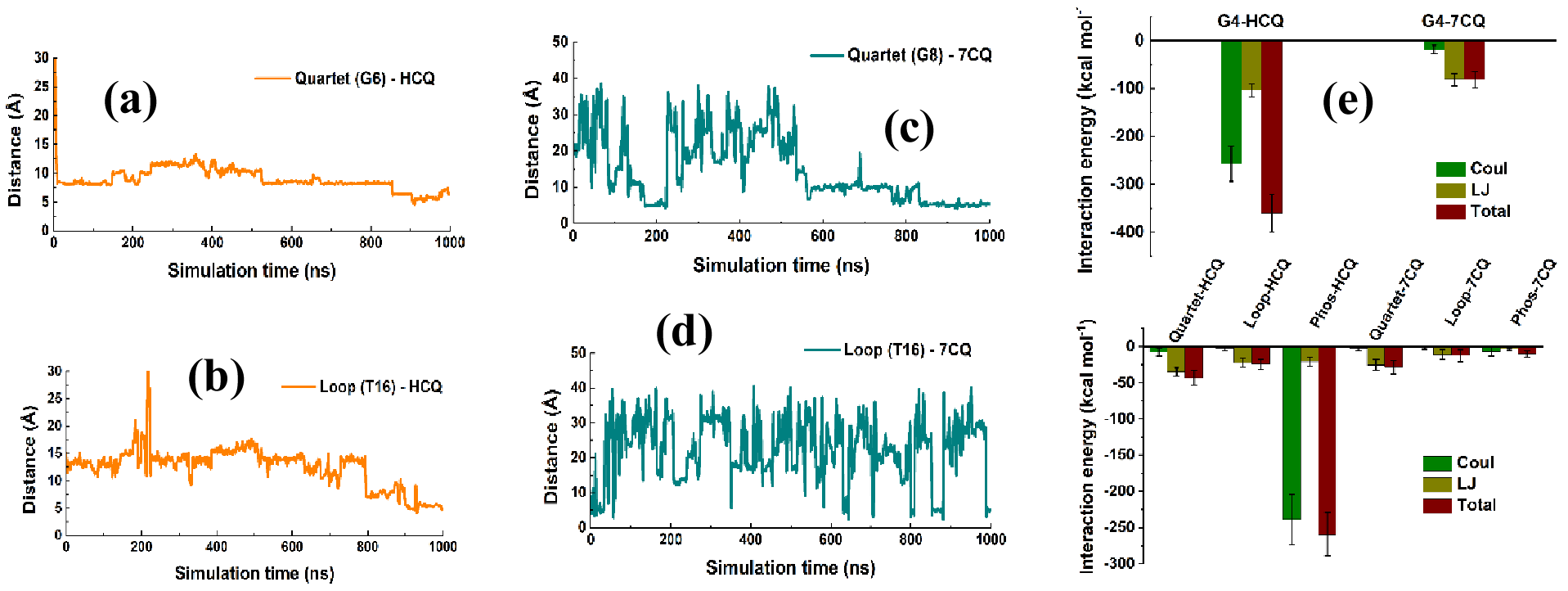


**Figure S15.** The distance variation for the quartet and loop bases of G4 with HCQ (a, b) and 7CQ (c, d), respectively for the entire simulation time scale. (e) The average total interaction energy of the HCQ and 7CQ molecules with the whole c-myc G4 structure. The total interaction energy is dissected into coulombic (Coul) and Lennard-Jones (LJ) terms (upper panel). The total and dissected interaction energies of the HCQ and 7CQ with the quartet, loop, and phosphate backbone of G4 (lower panel). Error bars indicate the standard deviation (SD) of the data.


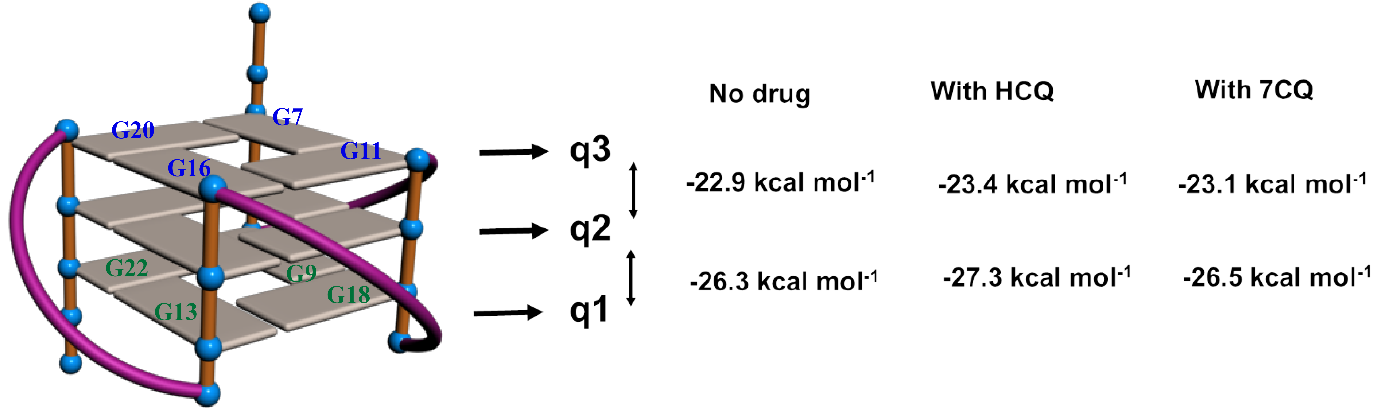


**Figure S16.** Schematic of the parallel G4 structure of c-myc with the representation of G bases involved in quartet 1 (q1), quartet 2 (q2), and quartet 3 (q3). The interaction energy between q1-q2 and q2-q3 in the absence of the drug and the presence of HCQ and 7CQ were represented. The atoms of the rings of the G bases involved in the quartet as shown in the schematic were selected for the interaction energy calculation.

**Table S1:** Sequence for ChIP-qRT-PCR

| **Gene Name** | **Sequence** |
| --- | --- |
| **c-myc** | F- CAGGACAAGGATGCGGGTTTG  R- TTTGCTCCCTCTCAAACCCT |
| **KRAS** | F- GTACGCCCGTCTGAAGAAGAA  R- CGAGCACACCGATGAGTTC |

**Table S2:** Sequence of primers for qRT-PCR analysis

| **Gene name** | **Forward(5`-3`)** | | **Reverse (5`-3`)** |
| --- | --- | --- | --- |
| **c-myc** | CCTGGTGCTCCATGAGGAGAC | CAGACTCTGACCTTTTGCCAGG | |
| **bcl2** | ATCGCCCTGTGGATGACTGAGT | GCCAGGAGAAATCAAACAGAGGC | |
| **KRAS** | CAGTAGACACAAAACAGGCTCG | TGTCGGATCTCCCTCACCAATG | |
| **c-kit** | CACCGAAGGAGGCACTTACACA | TGCCATTCACGAGCCTGTCGTA | |
| **VEGF** | TTGCCTTGCTGCTCTACCTCCA | GATGGCAGTAGCTGCGCTGATA | |
| **HK2** | GAGTTTGACCTGGATGTGGTTGC | CCTCCATGTAGCAGGCATTGCT | |
